# Omega-3 Fatty Acid Intake and Oxylipin Production in Response to Short-Term Ambient Air Pollution Exposure in Healthy Adults

**DOI:** 10.3390/toxics13121063

**Published:** 2025-12-09

**Authors:** Hao Chen, Siqi Zhang, Xiannen Pan, Alexandra Schneider, David Diaz-Sanchez, James Samet, Haiyan Tong

**Affiliations:** 1 Department of Occupational and Environmental Health, School of Public Health, Guangxi Medical University, Nanning 530021, China; 2 Oak Ridge Institute for Science and Education, Oak Ridge, TN 37831, USA; 3 Institute of Epidemiology, Helmholtz Zentrum München, 85764 Neuherberg, Germany; siqi.zhang@yale.edu (S.Z.); alexandra.schneider@helmholtz-munich.de (A.S.); 4 Department of Environmental Health Sciences, Yale School of Public Health, New Haven, CT 06511, USA; 5 Nursing College, Guangxi Medical University, Nanning 530021, China; 6 Public Health and Integrated Toxicology Division, Center for Public Health and Environmental Assessment, Office of Research and Development, U.S. Environmental Protection Agency, Research Triangle Park, NC 27711, USA

**Keywords:** ambient air pollution, oxylipins, omega-3 fatty acid, inflammation, inflammation resolution

## Abstract

Oxylipins are specialized lipid mediators that can have dual functions, either promoting inflammation or supporting resolution. Exposure to air pollution is associated with systemic inflammation that may be modified by oxylipins derived from polyunsaturated fatty acids (FA). In this study, we examined whether short-term air pollution exposure is associated with changes in circulating oxylipins in healthy adults, who were on high- or low-dietary omega-3 fatty acid (n-3 FA) intakes. We measured 56 oxylipin species from participants’ plasma samples and employed mixed-effects models to assess the associations, stratified by n-3 FA groups. Plasma concentrations of oxylipins derived from n-3 FA [e.g., 14-hydroxydocosahexaenoic acid (14-HDHA) & 11-hydroxydocosahexaenoic acid (11-HDoHE), and 12-hydroxyeicosapentaenoic acid (12-HEPE)] were significantly higher in the high n-3 FA group compared to the low group. Conversely, selected oxylipins derived from n-6 FA [e.g., 15-hydroxyeicosatetraenoic acid (15-HETE) and 14,15-Dihydroxyeicosatrienoic acid (14,15-DiHETrE)] were significantly lower in the high n-3 group. Exposure to PM_2.5_, O_3_, and NO_2_ was associated with reductions in pro-inflammatory oxylipins produced by lipoxygenase in the high n-3 FA group, but not in the low group; for example, 12-HETE. Furthermore, participants in the high n-3 group exposed to PM_2.5_, O_3_, and NO_2_ had elevated levels of n-3 FA-derived pro-resolving oxylipins compared to those in the low n-3 group; for instance, 12-HEPE and 14-HDHA & 11-HDoHE. In conclusion, short-term air pollution exposure was associated with lower pro-inflammatory and higher pro-resolving oxylipin levels in the high n-3 FA group. These findings suggest n-3-derived lipid metabolites may promote inflammation resolution induced by air pollution.

## 1. Introduction

Exposure to ambient particulate matter (PM), ozone (O_3_), and nitrogen dioxide (NO_2_) contributes to elevated morbidity and mortality caused by respiratory, cardiovascular, and neurological diseases [1,2,3,4,5,6,7,8,9,10]. Inflammation is believed to be a major response that underlies the adverse health effects of air pollution exposure. We have previously shown that exposure to ambient PM_2.5_ or O_3_ results in systemic inflammation that is attenuated by dietary intake of omega-3 fatty acids (n-3 FA) and exacerbated by that of omega-6 (n-6) FA [11]. The mechanisms involving FA-modulated inflammation in response to exposure to air pollution remain unexplored.

In general, n-3 FA metabolites have been shown to be anti-inflammatory, while those from n-6 FA are pro-inflammatory and can impair immune function [12]. The highly unsaturated long-chain FAs, docosahexaenoic acid (DHA), and eicosapentaenoic acid (EPA) are considered the most bioactive in the n-3 FA family, while arachidonic acid (AA) [13] and linoleic acid (LA) are major n-6 FAs. The inflammation and resolution processes influenced by n-3 and n-6 FAs occur through their metabolism to bioactive lipid mediators known as oxylipins. The biosynthesis of oxylipins occurs through oxidation of fatty acids, enzymatically by lipoxygenase (LOX), cyclooxygenase (COX), and cytochrome P450 (CYP), or through non-enzymatic free radical reactions such as lipid peroxidation [14]. Blood levels of oxylipins are produced in response to exogenous stimuli and are influenced by the activities of tissue-specific biosynthetic pathways and the availability of tissue n-3 and n-6 FA, which is largely determined by their dietary intake [14]. Shifts in mediator profiles subsequently drive physiological outcomes—spanning pro-inflammatory, anti-inflammatory, pro-resolving, and antioxidant functions [14]. The same enzymes generate oxylipins with antagonistic functions depending on the precursor. For instance, pro-inflammatory 12-hydroxyeicosatetraenoic acid (12-HETE) derived from AA through the LOX pathway is involved in the onset of the inflammatory response, while anti-inflammatory 13-hydroxyoctadecadienoic acid (13-HODE), also produced by the LOX pathway, is involved in the inflammation resolution process.

Previous evidence suggests that exposure to ambient air pollution is associated with altered levels of oxylipins in human blood [15,16,17,18,19]. A panel study conducted in Beijing, China, showed that blood levels of 12-HETE and 15-HETE were significantly associated with exposure to ambient PM_2.5_ [18]. Another study demonstrated that blood concentrations of 12-HETE decreased by 50% during the Beijing Olympics when air pollution levels were reduced, but increased by 119% when air pollution returned to pre-Olympics levels later in 2008 [19]. This study also observed a positive correlation between levels of 12-HETE and IL-8 during these time points [19]. Interestingly, young healthy adults traveling from Los Angeles to Beijing, where concentrations of PM_2.5_, O_3_, and NO_2_ were higher, experienced elevations in blood levels of 12-HETE and 15-HETE [20]. Controlled exposure to biodiesel exhaust resulted in altered plasma levels of monohydroxy fatty acids such as 12-HETE and 9-HODE in healthy adults [21]. Thus, existing evidence implicates oxylipins in air pollution-induced inflammation and resolution processes. However, whether n-3 fatty acid supplementation can shift oxylipin profiles toward a pro-resolving phenotype remains unexplored.

Given that availability of n-3 fatty acids determines anti-inflammatory and pro-resolving effects by modulating circulating oxylipins, this study tests the hypothesis that high blood n-3 FA levels modify the association between short-term ambient air pollution exposure and plasma oxylipin profiles in healthy human participants. By comparing participants stratified by n-3 FA status, we identify potential mechanisms whereby nutritional factors may mitigate pollution-induced oxylipin dysregulation.

## 2. Materials and Methods

### 2.1. Study Design and Study Subjects

This study is part of a larger panel study conducted in the Human Studies Facility (HSF) of the U.S. Environmental Protection Agency (EPA) in Chapel Hill, North Carolina, and registered with ClinicalTrials.gov (NCT02921048). As described previously [11,22], healthy adults aged 25–55 years old were enrolled in this study (Table 1). Dietary n-3 intake was quantitatively assessed using an in-house open-ended dietary questionnaire assessing fatty acid consumption over the past 6 months [23]. Participants also provided detailed records of fish oil supplement use. In addition, the erythrocyte levels of EPA, DHA, and other types of long-chain fatty acids were measured by gas chromatography, and the omega-3 index (EPA + DHA) was calculated (OmegaQuant, Sioux Falls, SD, USA). In this study, participants were categorized into low and high n-3 groups based on following criteria; low n-3 group: dietary EPA + DHA intake ≤ 0.5 g/week and omega-3 index ≤ 4%, high n-3 group: EPA + DHA intake ≥ 3 g/week and omega-3 index ≥ 5.5% [11,22,24]. All study participants gave informed consent. The Institutional Review Board at the University of North Carolina-Chapel Hill and U.S. EPA approved the protocol of this study.

**Figure 1:**
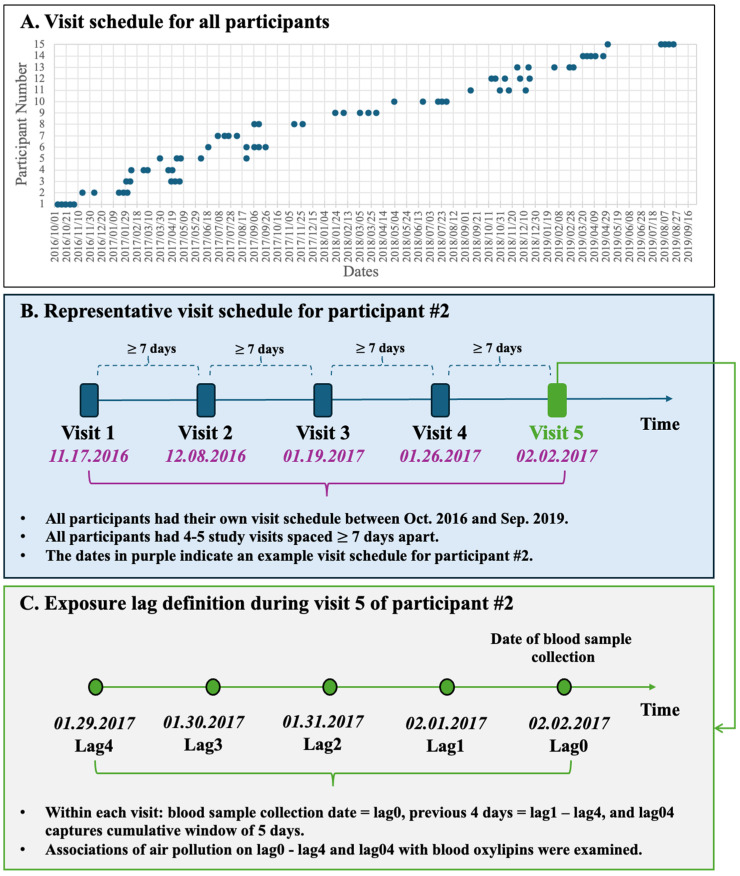
Study visits schedule and exposure lag structure. (**A**) Distribution of all 4–5 study visits for the 15 participants from October 2016 to September 2019. (**B**) Representative visit sequence for Participant #2; each visit was separated by ≥7 days. (**C**) Lag definitions for Participant #2’s fifth visit: blood-draw day = lag0, preceding 4 days = lag1–4, and the 5-day cumulative window = lag04.

Each participant had 4–5 repeat visits to HSF separated by at least 7 days between visits between October 2016 and September 2019 (Figure 1). We collected venous blood at each visit and plasma was stored at −80 °C prior to analysis. A portion of plasma was used for LC/MS/MS analysis, and another portion was used for inflammatory marker analysis. Commercially available multi-array plates were used to quantify levels of interleukin-6 (IL-6), IL-8, tumor necrosis factor alpha (TNFα), C-reactive protein (CRP), soluble intercellular adhesion molecule 1 (sICAM-1), and soluble vascular cell adhesion molecule 1 (sVCAM-1).

### 2.2. Exposure Assessment

Participants were recruited from within approximately a 97 km radius of the Research Triangle Park (RTP) area, and the central air quality monitor (Millbrook, Raleigh, NC, USA) used to assign exposures is located 44 km from the EPA HSF, where all blood draws were performed. All participants visited the HSF clinic on different days, but exposure windows were standardized to each individual’s visit date. As described previously [11,22], hourly levels of PM_2.5_, O_3_, and NO_2_ were obtained from the central air monitoring station and daily air-pollution levels were derived from hourly data (09:00 a.m.–08:00 a.m.): PM_2.5_ and NO_2_ were averaged over 24 h, and O_3_ was expressed as the daily maximum 8 h mean. Concurrent 24 h averages of temperature and relative humidity came from the same monitoring station. Each blood-draw visit was linked to exposure metrics for lag0 (the day of collection) and lag1–lag4 (the four preceding days), together with a cumulative 5-day moving average (lag04) (Figure 1).

### 2.3. Plasma Lipid Extraction and Purification

All standards and internal standards used for LC/MS/MS analysis of AA, DHA, EPA, and LA-derived oxylipins were purchased from Cayman Chemical (Ann Arbor, MI, USA). Plasma samples were pretreated for solid phase extraction (SPE) as described by Polinski et al. [25]. Briefly, proteins were precipitated from plasma with methanol and internal standard solution (5(S)-HETE-d_8_, 8-iso-PGF2a-d_4_, 9(S)-HODE-d_4_, LTB4-d_4_, LTD4-d_5_, LTE4-d_5_, PGE2-d_4_, PGF2a-d_9,_ and RvD2-d_5_ in ethanol). The samples were then centrifuged, and the supernatant was dried, and loaded onto Strata-X 33um SPE columns (Phenomenex, Torrance, CA, USA). Oxylipins were then eluted from columns. The samples were analyzed immediately or stored frozen at −70 °C until analyzed.

### 2.4. Liquid Chromatography–Tandem Mass Spectrometry

Reverse-phase HPLC tandem mass spectrometry system (LC/MS/MS) was performed for quantitation of oxylipins as described [25]. Briefly, the extracted sample was injected into an Agilent column, and mass spectrometric analysis was performed on an Agilent mass spectrometer (Agilent, Santa Clara, CA, USA). Oxylipins were quantified by dynamic Multiple Reaction Monitoring (MRM) after tuning collision energies via direct infusion of authentic standards; calibration curves spanned 0.25–250 pg on-column. An Agilent MassHunter Quantitative Analysis software (Version B.07.00) was employed to construct the calibration curves, which were further used to quantify lipid mediator concentrations in the plasma samples. A panel of 56 oxylipins was quantified from plasma samples, and 20 oxylipins were detectable at or above the limit of detection (LOD) thresholds (Table 2) and were ultimately included for further analysis in this study.

### 2.5. Statistical Analysis

The analyses were performed using R (version 3.6.2) with the “gamm4” and “mgcv” packages. Linear mixed-effects models (LMM) with random participant effects were used to examine the associations between air pollution with oxylipins in the high and low n-3 groups and test the between-group differences, as described [11]. LMMs were chosen to account for correlated repeated measures within participants. Model assumptions were verified through assessments of normality of residuals, homoscedasticity, and multicollinearity. For specific oxylipin levels detected below the LOD, we used the LOD values divided by square root of 2 for data analysis [26]. The statistical models were adjusted for covariates, including age, sex, race, marital status, education, and body mass index (BMI), long-term and seasonal trends, day of the week, temperature, and relative humidity. These covariates were selected because they may play a significant confounding effect [11,22]. Linear terms for daily O_3_, NO_2_, and PM_2.5_ were included to assess the interaction with groups separately. The immediate effects of air pollution on the visit day (lag0), delayed (lag1–lag4), and cumulative effects of 4 days (lag04) were investigated. In addition, we conducted preliminary pathway-enrichment analysis to identify possible biological pathways of oxylipins in response to air pollution exposure that were differentially abundant between low and high n-3 FA groups. Differential oxylipins were processed with MetaboAnalyst 5.0 [https://www.metaboanalyst.ca (accessed on 20 June 2025)]. Metabolite names were first standardized against the RaMP-DB database, after which pathway enrichment analyses were performed based on the differentially enriched metabolite sets between low and high n-3 FA groups. The top 10 most enriched pathways were selected and visualized as bubble plots. Spearman correlation analyses were also conducted for the relationships between circulating oxylipins and inflammation markers.

Robustness of the results was further assessed through several sensitivity analyses. First, we adjusted the analysis using two-pollutant models. Second, we excluded the adjustment for all individual characteristics in the regression model, considering the within-individual comparisons used for our repeatedly measured data. Third, we excluded outliers in the oxylipins, defined as the values exceeding three times the interquartile range (IQR) above the third quartile or below three times the IQR below the first quartile. Statistical significance was set at a two-sided *p* < 0.05 for the effects of air pollution and at a two-sided *p* < 0.1 for the interaction with n-3 FA groups.

## 3. Results

### 3.1. Participant Characteristics

The analysis included 15 participants who each visited the clinic 4–5 times at least 7 days apart for biomarker assessments. Among the 15 participants, seven (34 total visits) were classified into the low n-3 group, and eight (38 visits) were designated as the high n-3 group. The omega-3 index was 6.8 ± 1.6% in the high n-3 group and 3.8 ± 1.1% in the low n-3 group (*p* = 0.004). Baseline participant characteristics did not differ between the high and low n-3 groups, except for the higher mean age of participants in the high n-3 group (Table 1).

### 3.2. Ambient Air Pollution Measurements

The mean concentrations of 24 h-averaged PM_2.5_ and NO_2_ and daily maximum 8 h O_3_ during the study period are shown in Table 2. The concentrations of the three air pollutants were weakly to moderately correlated with each other (Spearman correlation coefficients ranged from −0.13 to 0.45). Throughout the study period, nearly all daily PM_2.5_ averages (98.6%), NO_2_ averages (100%), and maximum 8 h O_3_ averages (98.8%) remained below U.S. National Ambient Air Quality Standards [35 μg/m^3^ for PM_2.5_ (24-h), 100 ppb for NO_2_ (1 h), and 70 ppb for O_3_ (8-h), respectively] [https://www.epa.gov/criteria-air-pollutants/naaqs-table (accessed on 5 December 2025)]. Overall, ambient pollutant concentrations were comparable between the high- and low-n-3 groups.

### 3.3. Oxylipin Measurements

**Figure 2:**
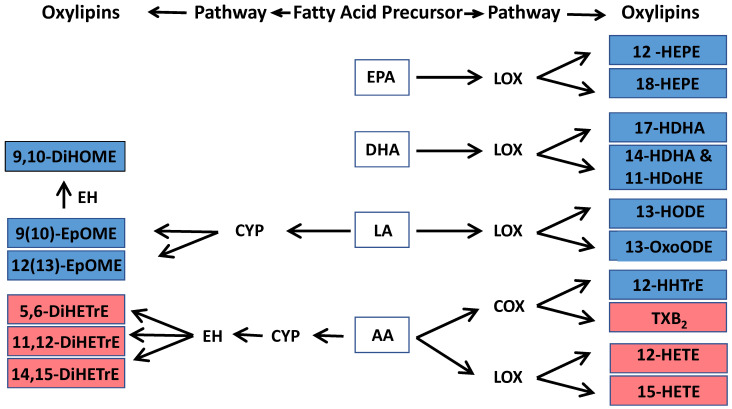
Oxylipins that were associated with low levels of ambient air pollution exposure in this study and their biosynthetic pathways. The oxylipin pathway is defined by a fatty acid precursor-eicosapentaenoic acid (EPA), docosahexaenoic acid (DHA), linoleic acid (LA), or arachidonic acid (AA) that is metabolized by an enzyme-lipoxygenase (LOX), cytochrome p450 (CYP), cyclooxygenase (COX), or epoxide hydrolase (EH). Notes: blue color indicates anti-inflammatory oxylipin, and red color represents pro-inflammatory oxylipin in response to air pollution exposure in this study. Complete names of oxylipins are provided in the list of abbreviations.

Twenty oxylipins detected above LOD thresholds were grouped by biosynthetic pathways from fatty acid precursors EPA, DHA, LA, and AA, and by enzyme/pathway groups, including LOX, COX, or CYP (Figure 2). Among these, 16 oxylipins were categorized into seven pathways: EPA-LOX, DHA-LOX, LA-CYP, LA-LOX, AA-COX, AA-CYP, and AA-LOX. Two oxylipins [9-HODE and 9-oxo-octadecadienoic acid (9-OxoODE)] produced by non-enzymatic reaction from LA were also detected. Another two oxylipins [15-hydroxyeicosatrienoic acid (15-HETrE)] are products of the dihomo-γ-linoleic acid (DGLA)-LOX pathway and 13-hydroxyoctadecatrienoic acid (13-HOTrE) in the α-linolenic acid (ALA)-LOX pathway (Table 3). We further classified these oxylipins into pro-inflammatory or pro-resolving mediators based on their responses to exposure to ambient air pollution (Figure 2).

Relative to the participants with low n-3 FA status, concentrations of pro-resolving oxylipins derived from DHA, including 14-HDHA & 11-HDoHE and 17-HDHA, were significantly higher (*p* < 0.05) in the high n-3 group. One notable exception is that the EPA-derived oxylipin 12-HEPE was markedly lower in the high n-3 group than in their low n-3 counterparts. In contrast, pro-inflammatory oxylipins, 15-HETE, and 14,15-DiHETrE, showed significantly lower concentrations in the high n-3 group in comparison to those in the low n-3 group (Table 3).

### 3.4. Overview of the Associations Between Exposure to Air Pollution and Oxylipins

In Table 4, we summarized differential associations between ambient air pollution exposure and circulating oxylipins in the low and high n-3 groups. Overall, circulating oxylipins remained unchanged with rising air-pollution levels among participants with low n-3 status. In contrast, those with high n-3 status mounted a pronounced response: pro-resolving oxylipins increased, and pro-inflammatory oxylipins decreased as pollutant concentrations rose. Detailed findings are presented below.

**Figure 3:**
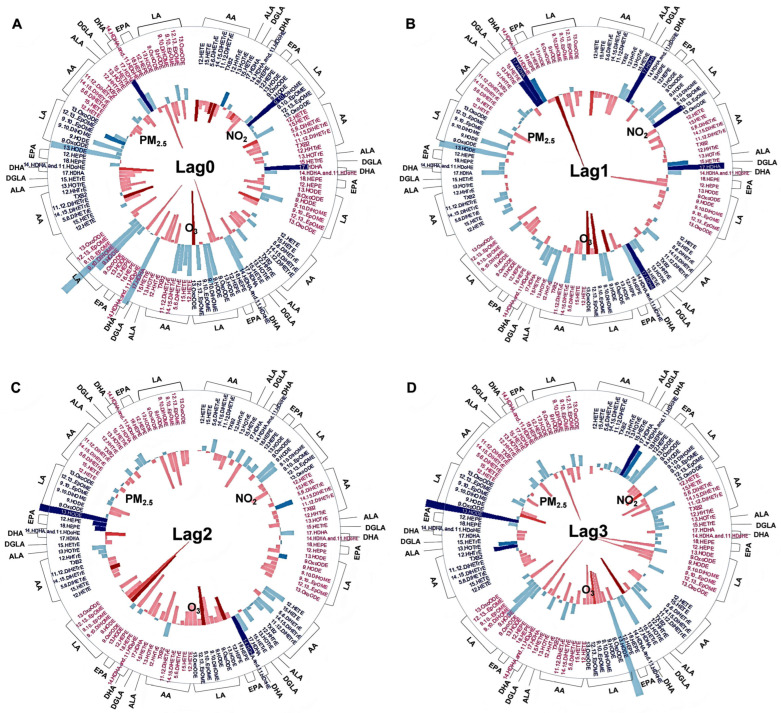

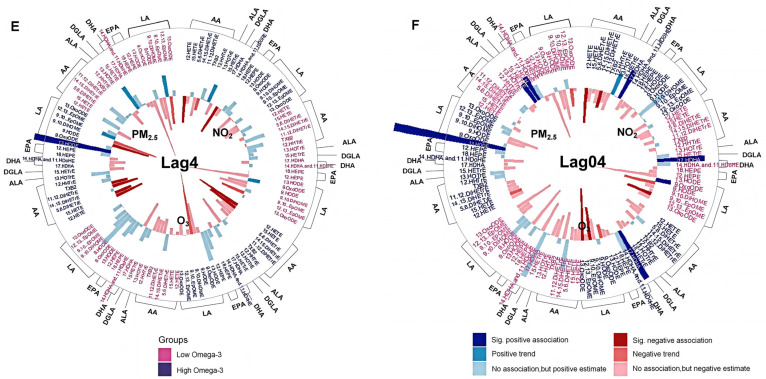
Association between short-term exposure to ambient PM_2.5,_ O_3,_ and NO_2_ and circulating oxylipins. Different colors on the oxylipin names indicate stratifications by omega-3 index (red: low n-3 group; dark blue: high n-3 group). The oxylipins were also grouped by their precursors, indicated on the outside of each wheel figure. The height of the bars indicates the percent change of each oxylipin in association with an IQR increase in ambient PM_2.5,_ O_3,_ and NO_2_ concentrations at lag0 day (**A**), lag1 day (**B**), lag2 day (**C**), lag3 day (**D**), lag4 day (**E**), and lag04 day (**F**); 5-day moving average from lag 0 to lag 4 day). The outward and inward directions of the bars indicate positive and negative association, respectively. Different levels of *p*-value are represented by colors. Sig. positive association: significant positive association with a *p*-value < 0.05. Positive trend: positive trend indicated 0.1 > *p*-value ≥ 0.05. Sig. negative association: significant negative association with a *p*-value < 0.05. Negative trend: negative trend indicated 0.1 > *p*-value ≥ 0.05. No association: no association indicated by *p*-value ≥ 0.1. AA: arachidonic acid, ALA: α-Linolenic acid, DGLA: dihomo-γ-linoleic acid, DHA: docosahexaenoic acid, EPA: eicosapentaenoic acid, LA: linoleic acid. Complete names of oxylipins are provided in the list of abbreviations.

### 3.5. Associations Between Exposure to Air Pollution and Altered Pro-Inflammatory Oxylipins

Among participants in the high n-3 group, higher ambient PM_2.5_ (Supplement Table S1), O_3_ (Supplement Table S2), and NO_2_ (Supplement Table S3) concentrations correlated with lower circulating pro-inflammatory oxylipins, and the associations were statistically stronger than the null associations in the low n-3 group (Figure 3). Among these oxylipins, levels of pro-inflammatory 12-HETE and 15-HETE were negatively associated with exposures to PM_2.5_ and O_3_ at lag4 day in the high n-3 group, with the associations stronger compared with those in the low n-3 group (Figure 3E). Similarly, the strength of the negative correlations between 12-HETE and NO_2_ exposure at lag0, lag4, and lag04 days in the high n-3 group were significantly stronger compared to the low n-3 group (Figure 3A,E,F).

Additionally, O_3_ exposure also significantly decreased levels of pro-inflammatory 9-OxoODE at lag1 day (Figure 3B) and reduced levels of 9(10)-EpOME at lag1, lag2, and lag04 days (Figure 3B,C,F) in the high n-3 group only.

### 3.6. Associations Between Exposure to Air Pollution and Altered Pro-Resolving Oxylipins

In contrast to the pro-inflammatory markers, several pro-resolving oxylipins were positively correlated with PM_2.5_ (Supplement Table S1), O_3_ (Supplement Table S2), and NO_2_ (Supplement Table S3) exposure in the high n-3 group, while no such associations were evident in the low n-3 group (Figure 3). Among these oxylipins, increased levels of 12-HEPE and 18-HEPE were significantly associated with PM_2.5_ exposure at lag2 day in the high n-3 group, and the associations were stronger compared to the null association in the low n-3 group (Figure 3C). Compared to the low n-3 group, levels of the pro-resolving mediator 13-HODE were significantly increased by PM_2.5_ exposure at lag2–4 and lag04 day in the high n-3 group compared to the low n-3 group (Figure 3C–F). In addition, levels of 13-HODE were decreased following PM_2.5_ exposure at lag1 day in the low n-3 group (Figure 3B). Levels of another pro-resolving mediator, 12(13)-epoxyoctadecanoic acid [12(13)-EpOME)], were increased significantly by NO_2_ exposure at lag1 day in the high n-3 group (Figure 3B). PM_2.5_ exposure was associated with significantly increased levels of anti-inflammatory 15-HETrE at lag1 and lag04 days in the low n-3 group, but these observations were not observed in the high group (Figure 3B,F). In contrast, levels of 9-HODE and 9,10-dihydroxyoctadecenoic acid (9,10-DiHOME) were reduced by O_3_ exposure at lag2 day in the low n-3 group (Figure 3C), while levels of 9-HODE and 9,10-DiHOME were decreased by PM_2.5_ exposure on later lag days in the high n-3 group (Figure 3D,E). The associations between air pollutants and oxylipins observed in the main analysis remained robust in two-pollutant models. Outliers of oxylipin data [i.e., 12-HETE (2 outliers), 15-HETE (3 outliers), 13-HODE (2 outliers), 11,12-DiHETrE (4 outliers), 12,13-DiHOME (4 outliers), 9,10-DiHOME (3 outliers), and 9-HODE (1 outlier)] were identified and excluded from the analysis and the associations remained stable.

### 3.7. Oxylipins, Biological Pathways, and Circulating Inflammatory Markers

**Figure 4:**
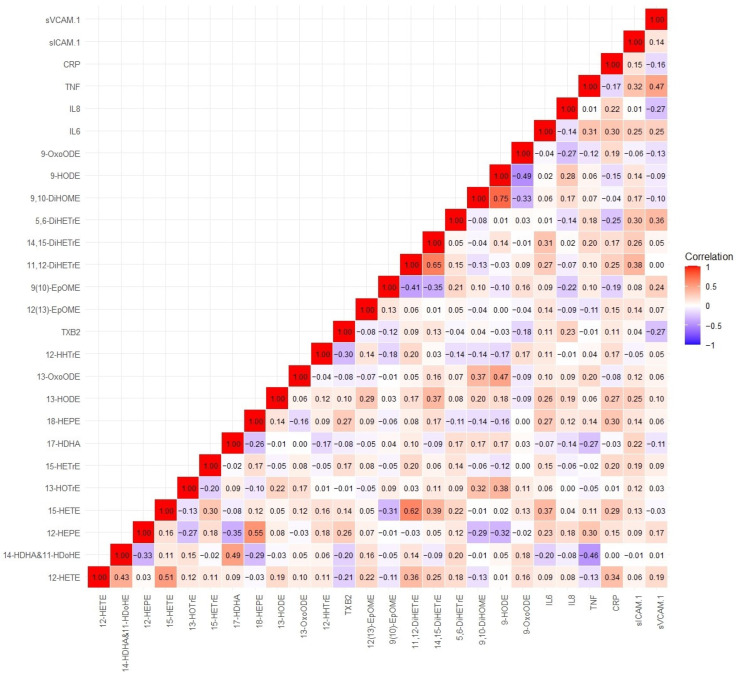
Spearman correlation coefficients between circulating oxylipins and inflammation markers. Complete names of oxylipins and inflammation markers are provided in the list of abbreviations.

**Figure 5:**
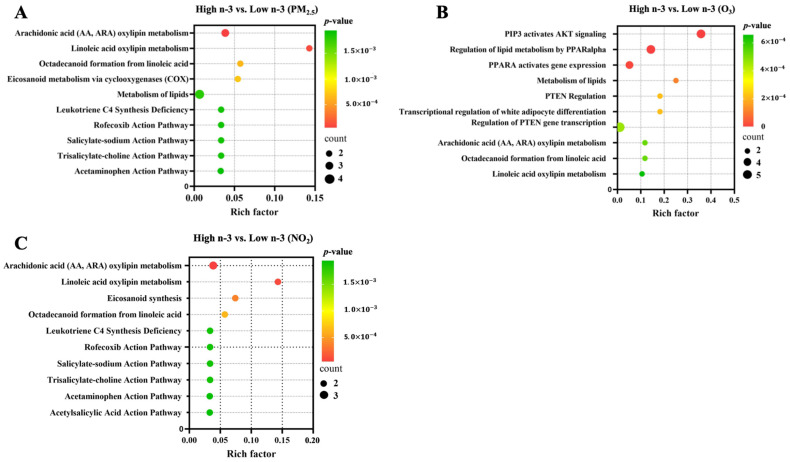
Biological pathways enriched based on significantly identified oxylipins in association with air pollutants between low and high n-3 FA groups. Top 10 enriched biological pathways were presented for (**A**) PM_2.5_, (**B**) O_3_, and (**C**) NO_2_, respectively. Differential oxylipins were processed with MetaboAnalyst 5.0 [https://www.metaboanalyst.ca (accessed on 20 June 2025)] to predict possible biological pathways with a Hypergeometric Test to calculate *p*-values.

Spearman correlation analyses between circulating oxylipins and inflammation markers are shown in Figure 4. Specifically, we observed a moderate negative correlation between 14-HDHA and 11-HDoHE with TNFα. There were weakly positive correlations between oxylipins and inflammatory markers, such as IL-6 with 11,12-DiHETrE, 14, 15-DiHETrE, and 15-HETE; CRP with 12-HETE, 15-HETE, 15-HETrE, and 11,12-DiHETrE. In addition, there were weak negative correlations between oxylipins and inflammatory markers, such as IL-6 with 14-HDHA and 11-HDoHE, and TNFα with 17-HDHA. Preliminary pathway enrichment analysis revealed distinct biological pathway profiles in response to air pollutants between low- and high-n-3 FA groups. Key pathways implicated in pollutant responses included: fatty acid metabolism (e.g., arachidonic acid metabolism), cellular signaling cascades (AKT activation), and transcriptional regulation (PPARα upregulation), suggesting n-3 FA status modulates fundamental biological responses to air pollution exposure (Figure 5).

## 4. Discussion

This study demonstrates that n-3 FA status significantly modifies the circulating lipid mediator response to short-term exposure to ambient air pollution in healthy volunteers. Specifically, among those with elevated n-3 PUFA levels, short-term increases in PM_2.5_, O_3_, and NO_2_ levels coincided with higher pro-resolving oxylipins yet lower pro-inflammatory oxylipins. These findings suggest that n-3-derived bioactive lipids may enable bioactive lipidomic adaptations in the direction of resolution-promoting phenotypes following low-level ambient air pollution exposure among healthy adults.

We have recently reported that plasma levels of n-3 FAs, including EPA, DHA, and DPA, were significantly elevated, while n-6 FAs (e.g., LA) and the ratio of AA/EPA and n-6/n-3 FAs were significantly reduced in participants who had higher intake of n-3 FAs [11]. We further showed that a higher n-3 FA index correlated with diminished cardiopulmonary responses to air pollution, whereas elevated n-6 FA intensified such a correlation, pointing to n-6 FA promoting air pollution-induced inflammation through oxidative stress signaling [11,22]. Beyond the global n-6/n3 ratio, the downstream fate of n-6 FA is critically shaped by gamma-linolenic acid (GLA) and its elongation product dihomo-gamma-linolenic acid (DGLA). GLA is synthesized from LA by Δ6-desaturase (D6D) and is subsequently elongated to DGLA, which is converted by 15-LOX to 15-HETrE—an eicosanoid with anti-inflammatory and LTB4-antagonistic properties [27,28]. Impaired D6D activity (genetic FADS2 variants, zinc/magnesium deficiency, chronic inflammation, or LA overload) limits GLA/DGLA formation and may shunt LA toward pro-inflammatory AA-derived mediators [29]. Interestingly, adding omega-3 fatty acids such as EPA to GLA-supplemented diets may prevent AA accumulation, a key pro-inflammatory n-6 FA [30]. However, in our study, we observed that short-term PM_2.5_ exposure was significantly associated with elevated plasma 15-HETrE in the low n-3 group but not in the high n-3 group, which may imply that adequate EPA/DHA status could also either spare DGLA from oxidative consumption or compensate D6D-mediated flux toward anti-inflammatory 15-HETrE. These data underscore the need for more research to investigate GLA together with EPA/DHA in future nutritional interventions targeting air-pollution-induced inflammation.

AA-derived 12-HETE and 15-HETE have been linked to airway inflammation, atherosclerosis, thrombogenesis, cardiovascular disease, metabolic disease, diabetes, and cancer [14,31,32,33]. These oxylipins may exert their actions through G protein-coupled receptor (GPCR)-signaling cascades [34]. Of note, air pollution exposure was significantly associated with plasma levels of 12-HETE and 15-HETE in several clinical trials [18,19,20,21]. Consistent with previous findings, our study demonstrates that plasma levels of 12-HETE were significantly reduced following short-term exposure to PM_2.5_, O_3_, or NO_2_ in the high n-3 group compared to those in the low n-3 group, suggesting that n-3 FA can attenuate the levels of 12-HETE induced by ambient air pollution exposure. 12-HETE acts as a potent neutrophil and leukocyte chemoattractant [35,36], triggering elevated expression of IL-6, TNFα, MCP1, and adhesion molecules in macrophages and vascular cells [36,37,38]. Consistent with its reported effects, we identified a positive correlation between 12-HETE and CRP, as well as 15-HETE and CRP or IL-6, suggesting that 12-HETE and 15-HETE mediate inflammatory processes and can be used as biomarkers of systemic inflammation after air pollution exposure.

Conversely, the hexeanoic acid products 17-HDHA and 14-HDHA, derived from DHA, and the pentaenoic acid metabolites, 12-HEPE and 18-HEPE, derived from EPA, exert anti-inflammatory effects by activating peroxisome proliferator-activated receptors (PPARs) [39], which were also identified in our pathway enrichment analysis. A panel study showed that 17-HDHA and 12-HEPE were positively associated with short-term moderate levels of PM_2.5_ exposure, suggesting that they mediate PM-induced systemic and cardiometabolic effects [18]. In the present study, 17-HDHA and 12-HEPE were significantly increased in response to O_3_, NO_2_, or PM_2.5_ exposure in the high n-3 group relative to the low n-3 group. We further demonstrate here that levels of 17-HDHA correlated negatively with blood concentrations of TNFα, suggesting that oxylipins derived from DHA and EPA may have protective effects against air pollution exposure through their anti-inflammatory effects.

Further, 9-HODE and 13-HODE, both LA derivatives, serve as oxidative stress indicators and constituents of the oxidized lipid complex within atherosclerotic plaque [40]. However, these oxylipins may have dual biological activities that are likely concentration- and context-dependent. It is suggested that early in the development of atherosclerosis, macrophage-derived 13-HODE, at lower concentrations, plays a protective role, enhancing the removal of lipids and cellular debris from the arterial wall through PPARγ activation. A study conducted during the Beijing Olympics, when stringent air pollution controls were in place, human plasma 13-HODE levels increased significantly but decreased non-significantly when the air pollutant levels rose later, indicating that 13-HODE may have pro-resolving properties [19]. However, later in the development of atherosclerotic lesions, increased concentrations of 9-HODE and 13-HODE mixtures generated by non-enzymatic oxidation of LA have adverse effects conducive to the onset of myocardial infarction and stroke through G-Protein Coupled Receptor (GPCR) signaling [40]. In our study, median 13-HODE levels (51–58 pg/mL) may well fall within the pro-resolving range, consistent with the observed inverse associations with CRP. The moderate elevations in 13-HODE were significantly associated with exposure to PM_2.5_ and O_3_ in the high n-3 group, suggesting that 13-HODE may play a pro-resolving role in the response to air pollution exposure. Interestingly, the levels of 9-HODE displayed a lag-time-dependent biphasic pattern, which is similar to our previous observations of other biomarkers [11,22], plausibly reflecting that 9-HODE has a protective effect following air pollution exposure early on but has a pro-inflammatory effect later in this study.

LA is converted to oxylipins through CYP metabolism to produce 9(10)-EpOME and 12(13)-EpOME, and these are further hydrolyzed by soluble epoxide hydrolase to 9,10-DiHOME and 12,13-DiHOME. EpOME and DiHOME may exert dose-dependent complex cardiovascular and respiratory effects: higher levels cause adverse effects, while lower doses may be protective [41,42]. The function of these metabolites in the alteration of immune responses and cardiopulmonary effects is mediated through PPARγ signaling and mitochondrial function [42]. It has been demonstrated that compared to healthy adults, 9,10-DiHOME level in the bronchoalveolar lavage fluid decreased in asthmatics exposed to pollutants found in subway air, suggesting that these oxylipins may have anti-inflammatory effects against ambient air pollution exposure [43]. In agreement, we found that plasma levels of 9,10-DiHOME and 12(13)-EpOME were lower in the low n-3 group, and levels of 12(13)-EpOME increased in the high n-3 group induced by exposure to air pollution, supporting the notion that 9,10-DiHOME and 12(13)-EpOME have pro-resolving effects of in healthy adults who had higher intake of n-3 FA.

Different xenobiotics may differentially induce redox stress through enzymatic and non-enzymatic pathways. Wood smoke has been reported to induce reactive oxygen species, possibly derived from lipid peroxidation and accumulation via aryl hydrocarbon receptor (AhR) activation [44]. In contrast, our low-level urban pollutant exposure appears to trigger enzymatic oxylipin remodeling. Similarly, perfluorooctanesulfonic acid (PFOS) exposure perturbs prostaglandin metabolism via a mechanism that involves Organic Anion Transporting Polypeptide (OATP) transporters [45], suggesting that different pollutants may target distinct segments of the oxylipin metabolism pathway. Diesel exhaust, rich in polycyclic aromatic hydrocarbons, can induce activation of CYP1A1 and AhR, leading to acute inflammation, altering detoxification enzymes, and implicating possible perturbations of oxylipin metabolism through the CYP pathways [46]. Our mixed-urban pollutant exposure likely represents a composite signature, with LOX-derived oxylipins showing the strongest responses. Additionally, long-term, continuous exposure to pollutants may lead to adaptive tolerance through epigenetic remodeling of LOX/COX promoters or, conversely, exhaustion of pro-resolving capacity if n-3 precursor availability is chronically insufficient. Future longitudinal studies are needed to determine whether these acute responses predict chronic disease trajectories.

The primary strength of this study is the repeated sampling of oxylipins in the plasma of the same individuals over time intervals of 1 week or more. Furthermore, we examined the alteration of oxylipins associated with low-level exposure to ambient air pollution, allowing us to apply the findings to real-world scenarios. Additionally, the assessment of the 56-oxylipin panel allowed us to examine changes in multiple pathways simultaneously. We have enriched pollutant-specific pathways based on the differentially identified oxylipins, suggesting n-3 FA levels may modulate lipid metabolism, inflammation, and oxidative stress-responsive pathways. However, we also recognize the limitations of this study. First, our sample size (n = 15 participants, 72 total visits) limits statistical power for detecting modest effects and may restrict generalizability to other sensitivity populations. However, the repeated-measures design increased our effective sample size and enabled robust within-person comparisons. Second, we used ambient air quality monitoring station data, which can introduce misclassification of personal exposure levels. However, our unpublished findings show a strong correlation between air pollution data from ambient air-quality-monitoring station data and individual exposure models for PM_2.5_ and moderate for O_3_, suggesting that ambient air-quality-monitoring station air pollution data are reasonable surrogates for such studies. Third, our BMI range (18.5–31.2 kg/m^2^) includes overweight participants, and it is possible that higher BMI levels may blunt oxylipin responses, warranting stratified investigation with a larger sample size in future studies. Finally, we only measured plasma oxylipins, which reflect acute biosynthesis, whereas erythrocyte membrane FAs integrate dietary intake over weeks, and future studies should examine erythrocyte and EV-derived oxylipins, which may be superior indicators of inflammation.

## 5. Conclusions

We observed significant novel associations between plasma oxylipins and short-term ambient air pollution exposure that were influenced by n-3 FAs. Levels of oxylipins with potential inflammatory properties were lower in the high n-3 FA group compared to the individuals in the low n-3 group following ambient air pollution exposure, which was most evident for oxylipins derived from AA. Conversely, potential anti-inflammatory and pro-resolving oxylipins were increased in the high n-3 group following air pollution exposure, with the most notable differences observed for oxylipins derived from EPA, DHA, and LA. These results provide preliminary evidence that n-3 FA-derived oxylipins may offer protection against inflammation induced by air pollution exposure, providing mechanistic insight into our previous findings that dietary intervention with n-3 FAs mitigates adverse health effects of air pollution exposure. Additionally, these n-3- and n-6-derived oxylipins may be used as surrogate markers of systemic inflammation induced by ambient air pollution exposure.

## Figures and Tables

**Table 1:** Participant characteristics at baseline.

Characteristics	All (n = 15)	Low n-3 Group (n = 7)	High n-3 Group (n = 8)	*p*
Age (years)	37 ± 10	31 ± 7	42 ± 9	0.03
BMI (kg/m^2^)	24.5 ± 3.1	24.0 ± 2.5	24.9 ± 3.7	0.56
Sex				0.99
Female	9 (60.0)	4 (57.1)	5 (62.5)	
Male	6 (40.0)	3 (42.9)	3 (37.5)	
Race				0.99
Caucasian	11 (73.3)	5 (71.4)	6 (75.0)	
African American	4 (26.7)	2 (28.6)	2 (25.0)	
Marital status				0.99
Single	9 (60.0)	6 (85.7)	3 (37.5)	
Married	4 (26.7)	0 (0)	4 (50.0)	
Separated/divorced	2 (13.3)	1 (14.3)	1 (12.5)	
Education				0.99
Graduate degree	5 (33.3)	2 (28.6)	3 (37.5)	
College degree	8 (53.3)	4 (57.1)	4 (50.0)	
High school/trade school	2 (13.3)	1 (14.3)	1 (12.5)	
Smoking history				
Nonsmoker	15 (100)	7 (100)	8 (100)	
SBP (mmHg)	110.8 ± 11.6	116.6 ± 7.5	105.8 ± 12.7	0.06
DBP (mmHg)	69.5 ± 8.9	71.3 ± 9.8	68.0 ± 8.4	0.38
Omega-3 index (%)	5.4 ± 2.1	3.8 ± 1.1	6.8 ± 1.6	0.004

Note: Values are expressed as either mean ± standard deviation or number (%). Low omega-3 (n-3) was defined as ≤0.5 g/week of eicosapentaenoic acid (EPA) + docosahexaenoic acid (DHA) intake for at least six months and an omega-3 index ≤ 4%; high n-3 was defined as ≥3 g/week of EPA + DHA intake for at least six months and an omega-3 index ≥ 5.5%. Seven participants contributed to 34 total visits and eight contributed to 38 visits in the low and high omega-3 groups, respectively. BMI: body mass index, DBP: diastolic blood pressure, SBP: systolic blood pressure.

**Table 2:** Air pollutant concentrations and meteorological measurements during the study period (6 October 2016–5 September 2019).

Exposure	Mean ± SD	Range	IQR
PM_2.5_ (µg/m^3^)	10.2 ± 4.1	1.8–68.0	4.7
O_3_ (ppb)	40.8 ± 11.1	10.0–71.0	17.0
NO_2_ (ppb)	5.3 ± 3.8	0.8–24.2	3.8
Temperature (°C)	16.5 ± 8.9	−8.6–31.1	15.2
Relative humidity (%)	70.2 ± 15.6	30.0–100.0	22.2

Note: IQR: interquartile range, NO_2_: nitrogen dioxide, O_3_: ozone, PM_2.5_: fine particulate matter, ppb: parts per billion, SD: standard deviation.

**Table 3:** Average concentrations of circulating oxylipins detected in this study.

Oxylipin (pg/mL)	Precursor Fatty Acid	Pathway	All	Low n-3 Group (n = 34)	High n-3 Group (n = 38)	*p* Value
12-HETE	AA	LOX	384.0 ± 351.0	458.0 ± 459.0	318.0 ± 196.0	0.091
14-HDHA & 11-HDoHE	DHA	LOX	17.5 ± 15.1	11.3 ± 13.1	23.1 ± 14.8	<0.001 *
12-HEPE	EPA	LOX	32.0 ± 13.6	37.4 ± 8.4	27.2 ± 15.5	0.001 *
15-HETE	AA	LOX	182.0 ± 92.0	208.0 ± 115.0	158.0 ± 58.0	0.021 *
13-HOTrE	ALA	LOX	36.5 ± 20.9	31.6 ± 20.3	40.9 ± 20.7	0.059
15-HETrE	DGLA	LOX	21.8 ± 11.4	22.3 ± 12.9	21.3 ± 10.1	0.714
17-HDHA	DHA	LOX	10.1 ± 9.8	6.0 ± 7.2	13.8 ± 10.3	<0.001 *
18-HEPE	EPA	LOX	32.5 ± 12.0	35.4 ± 0.0	30.0 ± 16.3	0.058
13-HODE	LA	LOX	54.2 ± 112.9	57.6 ± 116.4	51.1 ± 111.2	0.809
13-OxoODE	LA	LOX	36.5 ± 20.9	36.3 ± 22.4	36.6 ± 19.7	0.952
12-HHTrE	AA	COX	32.7 ± 18.3	33.7 ± 18.6	31.8 ± 18.3	0.664
TXB_2_	AA	COX	37.2 ± 23.8	39.6 ± 21.8	35.1 ± 25.5	0.426
12(13)-EpOME	LA	CYP	33.3 ± 18.1	35.4 ± 19.0	31.5 ± 17.4	0.366
9(10)-EpOME	LA	CYP	28.2 ± 13.5	25.5 ± 14.6	30.6 ± 12.2	0.111
11,12-DiHETrE	AA	EH	190.0 ± 82.0	203.0 ± 106.0	179.0 ± 52.0	0.219
14,15-DiHETrE	AA	EH	36.5 ± 20.9	42.4 ± 22.2	31.2 ± 18.4	0.022 *
5,6-DiHETrE	AA	EH	30.3 ± 15.0	27.0 ± 12.7	33.3 ± 16.4	0.075
9,10-DiHOME	LA	EH	6484.0 ± 7614.0	5622.0 ± 5016.0	7255.0 ± 9355.0	0.367
9-HODE	LA	NA	6036.0 ± 5313.0	5796.0 ± 5343.0	6251.0 ± 5349.0	0.720
9-OxoODE	LA	NA	30.9 ± 10.7	28.4 ± 12.8	33.1 ± 8.0	0.063

Note: Values are expressed as mean ± standard deviation. Low n-3 group with n-3 index ≤ 4% and high n-3 group with n-3 index ≥ 5.5%. A t-test was applied to examine the difference in oxylipin concentrations between low and high n-3 groups. * *p* < 0.05 indicates statistical significance. See the abbreviation list for the full name of oxylipins, precursor fatty acids, and pathway enzymes.

**Table 4:** Summary of associations between air pollutants and circulating oxylipin in low and high n-3 groups.

Precursors	Oxylipins	Key Function	PM_2.5_		O_3_		NO_2_	
			Low n-3	High n-3	Low n-3	High n-3	Low n-3	High n-3
AA	12-HHTrE	Pro-resolution	→	↑_lag4_	→	→	→	→
	12-HETE	Pro-inflammation	→	↓_lag4_	→	↓_lag4_	→	↓_lag0, lag4, lag04_
	15-HETE	Pro-inflammation	→	↓_lag4_	→	↓_lag4_	→	→
	5,6-DiHETrE	Pro-inflammation	→	↓_lag0, lag2_	→	→	→	→
	11,12-DiHETrE	Pro-inflammation	→	↓_lag4_	→	→	→	↓_lag0, lag04_
	14,15-DiHETrE	Pro-inflammation	→	↓_lag4_	→	→	→	→
	TXB_2_	Pro-inflammation	→	→	→	→	→	↓_lag0_
DGLA	15-HETrE	Anti-inflammation	↑_lag1, lag04_	→	→	→	→	→
LA	9-HODE	Pro-inflammation	→	↓_lag3, lag4_	↓_lag2_	→	→	→
	13-HODE	Pro-resolution	↓_lag1_	↑_lag2–4, lag04_	→	→	→	→
	9-OxoODE	Pro-resolution	→	→	→	↓_lag1_	↑_lag04_	→
	13-OxoODE	Pro-resolution	→	→	→	↓_lag0–1, lag04_	→	→
	9,10-DiHOME	Pro-resolution	→	↓_lag4_	↓_lag2_	→	→	↑_lag0_
	9(10)-EpOME	Pro-resolution	→	→	→	↓_lag1–2, lag04_	→	→
	12(13)-EpOME	Pro-resolution	→	→	→	→	↓_lag04_	↑_lag1_
ALA	13-HOTrE	Pro-resolution	→	→	→	→	→	→
DHA	14-HDHA and 11-HDoHE	Pro-resolution	→	→	→	↑_lag2_	→	→
	17-HDHA	Pro-resolution	↑_lag0–1, lag04_	→	→	↑_lag04_	↑_lag0–1, lag04_	↑_lag1, lag3, lag04_
EPA	12-HEPE	Pro-resolution	→	↑_lag2_	→	↓_lag2_	→	→
	18-HEPE	Pro-resolution	→	↑_lag2_	→	→	→	→

Note: Arrows “↓, ↑, and →” indicate negative, positive, and null associations between air pollutant and cardiovascular biomarker, respectively. Blue color indicates anti-inflammatory or pro-resolution oxylipin, and red color represents pro-inflammatory oxylipin in response to air pollution exposure in this study. Detailed data are available in Figure 3 and Supplemental Tables S1–S3. See the abbreviation list for the full name of oxylipins and precursor fatty acids.

## Data Availability

The data presented in this study are available in the public accessible depository, the EPA ScienceHub (https://catalog.data.gov/dataset/epa-sciencehub), after the paper is published.

## Supplementary Materials

The following supporting information can be downloaded at: https://www.mdpi.com/article/10.3390/toxics13121063/s1. Supplemental Table S1. Associations between PM_2.5_ exposure and changes in blood levels of oxylipins. Supplemental Table S2. Associations between O_3_ exposure and changes in blood levels of oxylipins. Supplemental Table S3. Associations between NO_2_ exposure and changes in blood levels of oxylipins.

## Abbreviations

AA	arachidonic acid
ALA	α-Linolenic acid
CRP	C-reactive protein
COX	cyclooxygenase
CYP	cytochrome p450
DGLA	dihomo-γ-linoleic acid
DHA	docosahexaenoic acid
DiHETrE	dihydroxyeicosa-trienoic acid
DiHOME	dihydroxyoctadecenoic acid
DPA	docosapentaenoic acid
EPA	eicosapentaenoic acid
EpOME	epoxyoctadecenoic acid
HDHA	hydroxydocosahexaenoic acid
HEPE	hydrooxyeicosapentaenoic acid
HETE	hydroxyeicosatetraenoic acid
HETrE	hydroxyeicosatrienoic acid
HHTrE	hydroxyheptadecatrienoic acid
HODE	hydroxyoctadecadienoic acid
HOTrE	hydroxyoctadecatrienoic acid
IL	interleukin
LA	linoleic acid
LOX	lipoxygenase
MRM	Multiple Reaction Monitoring
n-3 FA	omega-3 polyunsaturated fatty acid
n-6 FA	omega-6 polyunsaturated fatty acids
OxoODE	oxooctadecadienoic acid
PPAR	peroxisome proliferator-activated receptors
sEH	soluble epoxide hydrolase
sICAM1	soluble intercellular adhesion molecule-1
TNFα	tumor necrosis factor alpha
TXB2	thromboxane B2
