# Supplementary material for: Omega-3 Fatty Acid Intake and Oxylipin Production in Response to Short-Term Ambient Air Pollution Exposure in Healthy Adults
Source: Toxics. 2025 Dec 9;13(12):1063. doi: 10.3390/toxics13121063 (PMC12737357; doi:10.3390/toxics13121063)
Supplement: Supplementary file 1 [file toxics-13-01063-s001.zip › toxics-3927328-supplementary.pdf]

SUPPLEMENTAL MATERIALS

Omega-3 Fatty Acid Intake and Oxylipin Production in Response to Short-Term Ambient Air Pollution Exposure in Healthy Adults

Hao Chen [1,2,*], Siqi Zhang [3,4], Xiannen Pan [5], Alexandra Schneider [3], David Diaz-Sanchez [6], James Samet [6] and Haiyan Tong [6,*]

[1] Department of Occupational and Environmental Health, School of Public Health, Guangxi Medical University, Nanning 530021, China
[2] Oak Ridge Institute for Science and Education, Oak Ridge, TN 37831, USA
[3] Institute of Epidemiology, Helmholtz Zentrum München, 85764 Neuherberg, Germany; siqi.zhang@yale.edu (S.Z.); alexandra.schneider@helmholtz-munich.de (A.S.)
[4] Department of Environmental Health Sciences, Yale School of Public Health, New Haven, CT 06511, USA
[5] Nursing College, Guangxi Medical University, Nanning 530021, China
[6] Public Health and Integrated Toxicology Division, Center for Public Health and Environmental Assessment, Office of Research and Development, U.S. Environmental Protection Agency, Research Triangle Park, NC 27711, USA
* Correspondence: hchen255@gmail.com (H.C.); tong.haiyan@epa.gov (H.T.); Tel.: +86-0771-5350823 (H.C.); +86-(919)-541-5572 (H.T.)

Supplemental Table S1. Associations between $PM_{2.5}$ exposure and changes in blood levels of oxylipins.

Oxylipins (pg/mL)	Exposure	Lag	Low n-3 group	P value	High n-3 group	P value	P value between groups
12-HETE	$PM_{2.5}$	lag0	-3 (-37.5, 31.5)	0.87	7 (-33.1, 47.2)	0.73	0.73
12-HETE	$PM_{2.5}$	lag1	5.1 (-28.8, 38.9)	0.77	13.7 (-17.4, 44.8)	0.39	0.39
12-HETE	$PM_{2.5}$	lag2	7.2 (-24, 38.3)	0.65	-9.9 (-35.3, 15.5)	0.44	0.44
12-HETE	$PM_{2.5}$	lag3	-14 (-51.2, 23.2)	0.47	-14.4 (-35, 6.1)	0.17	0.17
12-HETE	$PM_{2.5}$	lag4	22.3 (-10.6, 55.3)	0.19	**-32.4 (-52, -12.8)**	**<0.01**	**<0.01**
12-HETE	$PM_{2.5}$	lag04	6.8 (-41.4, 54.9)	0.78	-26.9 (-63.4, 9.6)	0.15	0.15
15-HETE	$PM_{2.5}$	lag0	-4.3 (-27.6, 19.1)	0.72	-19 (-47.9, 9.8)	0.20	0.20
15-HETE	$PM_{2.5}$	lag1	-1.2 (-22.9, 20.5)	0.91	8.9 (-14.7, 32.4)	0.46	0.46
15-HETE	$PM_{2.5}$	lag2	-8 (-31.2, 15.2)	0.50	-10.6 (-29.3, 8)	0.26	0.26
15-HETE	$PM_{2.5}$	lag3	1.8 (-23.9, 27.4)	0.89	-9.8 (-26.2, 6.6)	0.24	0.24
15-HETE	$PM_{2.5}$	lag4	0 (-24.6, 24.5)	1.00	**-20 (-36.8, -3.3)**	**0.02**	**0.02**
15-HETE	$PM_{2.5}$	lag04	-6.9 (-38.3, 24.6)	0.67	-25.9 (-54.9, 3)	0.08	0.08
12-HHTrE	$PM_{2.5}$	lag0	-9.6 (-40.7, 21.4)	0.55	-12 (-49.7, 25.7)	0.53	0.53
12-HHTrE	$PM_{2.5}$	lag1	3.8 (-23.2, 30.8)	0.79	-2.9 (-35.5, 29.7)	0.86	0.86
12-HHTrE	$PM_{2.5}$	lag2	-11.3 (-43.6, 21)	0.50	12.3 (-13.9, 38.5)	0.36	0.36
12-HHTrE	$PM_{2.5}$	lag3	-18.7 (-51.8, 14.4)	0.27	**26.1 (4.6, 47.6)**	**0.02**	**0.02**
12-HHTrE	$PM_{2.5}$	lag4	-16.9 (-52.7, 18.9)	0.36	4.8 (-20, 29.7)	0.70	0.70
12-HHTrE	$PM_{2.5}$	lag04	-12.7 (-55.6, 30.2)	0.56	22.1 (-19.5, 63.7)	0.30	0.30
5,6-DiHETrE	$PM_{2.5}$	lag0	-10.6 (-30.8, 9.5)	0.31	**-35.9 (-62, -9.8)**	**0.01**	**0.01**
5,6-DiHETrE	$PM_{2.5}$	lag1	2.6 (-16.8, 22.1)	0.79	-3 (-25.5, 19.5)	0.80	0.80
5,6-DiHETrE	$PM_{2.5}$	lag2	2 (-14.3, 18.4)	0.81	**-15 (-28.5, -1.6)**	**0.03**	**0.03**
5,6-DiHETrE	$PM_{2.5}$	lag3	-3.3 (-26.8, 20.2)	0.78	-10.6 (-25.4, 4.3)	0.16	0.16
5,6-DiHETrE	$PM_{2.5}$	lag4	10.4 (-13.7, 34.4)	0.40	3.2 (-13.4, 19.8)	0.71	0.71
5,6-DiHETrE	$PM_{2.5}$	lag04	-0.6 (-29.7, 28.6)	0.97	-23.1 (-51.5, 5.3)	0.11	0.11
11,12-DiHETrE	$PM_{2.5}$	lag0	9.2 (-12, 30.4)	0.40	-11.5 (-37.1, 14.2)	0.38	0.38
11,12-DiHETrE	$PM_{2.5}$	lag1	13 (-4.7, 30.7)	0.16	9.4 (-11.9, 30.8)	0.39	0.39
11,12-DiHETrE	$PM_{2.5}$	lag2	-10.7 (-31.6, 10.2)	0.32	-12.2 (-29.2, 4.7)	0.16	0.16
11,12-DiHETrE	$PM_{2.5}$	lag3	5.8 (-17.6, 29.1)	0.63	-3.4 (-18.6, 11.8)	0.66	0.66
11,12-DiHETrE	$PM_{2.5}$	lag4	3 (-20, 26.1)	0.80	**-18.5 (-34.5, -2.5)**	**0.02**	**0.02**
11,12-DiHETrE	$PM_{2.5}$	lag04	10.7 (-17.9, 39.3)	0.47	-18 (-46, 10)	0.21	0.21
14,15-DiHETrE	$PM_{2.5}$	lag0	-8.2 (-42, 25.6)	0.64	2.4 (-37.7, 42.5)	0.91	0.91
14,15-DiHETrE	$PM_{2.5}$	lag1	3.5 (-24.5, 31.5)	0.81	18 (-15.6, 51.6)	0.29	0.29
14,15-DiHETrE	$PM_{2.5}$	lag2	-7.1 (-41.8, 27.6)	0.69	-12.1 (-39.8, 15.7)	0.39	0.39
14,15-DiHETrE	$PM_{2.5}$	lag3	-0.1 (-40.9, 40.6)	0.99	-7.5 (-31.9, 16.8)	0.55	0.55
14,15-DiHETrE	$PM_{2.5}$	lag4	22.3 (-15.1, 59.7)	0.25	**-29.8 (-55.4, -4.2)**	**0.02**	**0.02**
14,15-DiHETrE	$PM_{2.5}$	lag04	0.3 (-46.8, 47.3)	0.99	-19.4 (-63.2, 24.4)	0.39	0.39
TXB2	$PM_{2.5}$	lag0	4.7 (-25.7, 35.1)	0.76	-28.9 (-65, 7.1)	0.12	0.12
TXB2	$PM_{2.5}$	lag1	4.6 (-21.8, 31.1)	0.73	-2.9 (-34.7, 28.9)	0.86	0.86
TXB2	$PM_{2.5}$	lag2	12.1 (-19, 43.3)	0.45	8.2 (-16.8, 33.1)	0.52	0.52
TXB2	$PM_{2.5}$	lag3	11.7 (-25.3, 48.8)	0.54	2.4 (-19.7, 24.6)	0.83	0.83
TXB2	$PM_{2.5}$	lag4	2.3 (-35, 39.5)	0.91	10.5 (-15, 36)	0.42	0.42
TXB2	$PM_{2.5}$	lag04	11.4 (-32.8, 55.6)	0.62	1.7 (-39.5, 42.8)	0.94	0.94
15-HETrE	$PM_{2.5}$	lag0	21 (-8.2, 50.2)	0.17	-18.1 (-53.3, 17)	0.31	0.31
15-HETrE	$PM_{2.5}$	lag1	**37.7 (15.2, 60.2)**	**0.002**	-3.8 (-30.9, 23.3)	0.78	0.78
15-HETrE	$PM_{2.5}$	lag2	14.3 (-16.4, 45)	0.37	-10.1 (-34.6, 14.5)	0.42	0.42
15-HETrE	$PM_{2.5}$	lag3	12 (-25.1, 49.1)	0.53	-7.8 (-29.4, 13.9)	0.48	0.48
15-HETrE	$PM_{2.5}$	lag4	30.3 (-4.8, 65.4)	0.10	-16.8 (-40.5, 6.9)	0.17	0.17
15-HETrE	$PM_{2.5}$	lag04	**47.9 (9, 86.8)**	**0.02**	-23.5 (-59.5, 12.5)	0.20	0.20
9-HODE	$PM_{2.5}$	lag0	-25.1 (-62.1, 11.9)	0.19	35.2 (-10.7, 81.1)	0.13	0.13
9-HODE	$PM_{2.5}$	lag1	-15.9 (-54.5, 22.6)	0.42	35.1 (-7.1, 77.3)	0.10	0.10
9-HODE	$PM_{2.5}$	lag2	-29.3 (-70.5, 11.9)	0.17	-19.1 (-52.2, 13.9)	0.26	0.26
9-HODE	$PM_{2.5}$	lag3	-7.3 (-54.1, 39.4)	0.76	**-28.9 (-58.1, 0.3)**	**0.05**	**0.05**
9-HODE	$PM_{2.5}$	lag4	-24.9 (-67.5, 17.7)	0.26	**-43.2 (-72.1, -14.2)**	**0.004**	**0.004**
9-HODE	$PM_{2.5}$	lag04	-41.4 (-98.5, 15.6)	0.16	-38.5 (-90.4, 13.5)	0.15	0.15
13-HODE	$PM_{2.5}$	lag0	-54.1 (-157.5, 49.4)	0.31	81.9 (-52.1, 216)	0.23	0.23
13-HODE	$PM_{2.5}$	lag1	**-102.7 (-184.6, -20.9)**	**0.02**	85.2 (-18.8, 189.2)	0.11	0.11
13-HODE	$PM_{2.5}$	lag2	-53.6 (-153.3, 46.1)	0.30	**105.3 (20.3, 190.3)**	**0.02**	**0.02**
13-HODE	$PM_{2.5}$	lag3	-57.7 (-163.3, 47.9)	0.29	**102 (33, 171)**	**0.00**	**0.00**
13-HODE	$PM_{2.5}$	lag4	-67.7 (-181.4, 46)	0.25	**89 (9, 168.9)**	**0.03**	**0.03**
13-HODE	$PM_{2.5}$	lag04	-101 (-221.7, 19.6)	0.11	**241.2 (114.3, 368.1)**	**0.00**	**0.00**
9-OxoODE	$PM_{2.5}$	lag0	7.6 (-3.8, 19)	0.20	6.1 (-8.3, 20.5)	0.41	0.41

Supplemental Table 1 Continued

9-OxoODE	$PM_{2.5}$	lag1	0.9 (-13.1, 14.8)	0.90	-8.6 (-23.4, 6.1)	0.25	0.25
9-OxoODE	$PM_{2.5}$	lag2	4 (-10.5, 18.6)	0.59	3.7 (-7.9, 15.4)	0.53	0.53
9-OxoODE	$PM_{2.5}$	lag3	4.7 (-12, 21.4)	0.58	2.9 (-7.4, 13.1)	0.59	0.59
9-OxoODE	$PM_{2.5}$	lag4	11.4 (-4.2, 27)	0.16	6.5 (-4.1, 17.1)	0.23	0.23
9-OxoODE	$PM_{2.5}$	lag04	9.7 (-10.6, 30.1)	0.36	6.1 (-12.4, 24.6)	0.52	0.52
13-OxoODE	$PM_{2.5}$	lag0	13.1 (-23.2, 49.4)	0.48	1.6 (-40.8, 44.1)	0.94	0.94
13-OxoODE	$PM_{2.5}$	lag1	-6.9 (-36.7, 22.9)	0.65	-22.8 (-58.9, 13.4)	0.22	0.22
13-OxoODE	$PM_{2.5}$	lag2	-21.5 (-57.7, 14.8)	0.25	-2.7 (-32.4, 26.9)	0.86	0.86
13-OxoODE	$PM_{2.5}$	lag3	11.8 (-27.7, 51.4)	0.56	6.6 (-19.7, 33)	0.62	0.62
13-OxoODE	$PM_{2.5}$	lag4	6 (-34, 46.1)	0.77	-16.8 (-44.7, 11)	0.24	0.24
13-OxoODE	$PM_{2.5}$	lag04	-7.2 (-55.9, 41.5)	0.77	-16.1 (-64.6, 32.5)	0.52	0.52
9,10-DiHOME	$PM_{2.5}$	lag0	-16.8 (-86.3, 52.8)	0.64	3.1 (-78.3, 84.5)	0.94	0.94
9,10-DiHOME	$PM_{2.5}$	lag1	-31 (-89.6, 27.5)	0.31	2.1 (-69, 73.1)	0.95	0.95
9,10-DiHOME	$PM_{2.5}$	lag2	-51.7 (-121, 17.6)	0.15	-31.1 (-87.9, 25.6)	0.28	0.28
9,10-DiHOME	$PM_{2.5}$	lag3	-16.2 (-94.7, 62.3)	0.69	-18 (-70.2, 34.1)	0.50	0.50
9,10-DiHOME	$PM_{2.5}$	lag4	-38.5 (-113.6, 36.5)	0.32	**-51.8 (-104, 0.5)**	**0.05**	**0.05**
9,10-DiHOME	$PM_{2.5}$	lag04	-65.1 (-156.7, 26.6)	0.17	-65.8 (-156.9, 25.4)	0.16	0.16
9(10)-EpOME	$PM_{2.5}$	lag0	18.3 (-4.4, 41)	0.12	25 (-3.1, 53.1)	0.08	**0.08**
9(10)-EpOME	$PM_{2.5}$	lag1	6.3 (-16.8, 29.4)	0.60	-5.6 (-31.2, 19.9)	0.66	0.66
9(10)-EpOME	$PM_{2.5}$	lag2	0.3 (-24.7, 25.3)	0.98	7.4 (-12.7, 27.5)	0.47	0.47
9(10)-EpOME	$PM_{2.5}$	lag3	14.3 (-13.9, 42.4)	0.33	-0.7 (-18.6, 17.3)	0.94	0.94
9(10)-EpOME	$PM_{2.5}$	lag4	24.5 (-1.7, 50.8)	0.07	15.7 (-2.2, 33.6)	0.09	**0.09**
9(10)-EpOME	$PM_{2.5}$	lag04	20.4 (-13.8, 54.7)	0.25	18.5 (-13.2, 50.2)	0.25	0.25
12(13)-EpOME	$PM_{2.5}$	lag0	-22 (-51.2, 7.2)	0.15	15 (-20.5, 50.4)	0.41	0.41
12(13)-EpOME	$PM_{2.5}$	lag1	-18.2 (-43.3, 7)	0.17	18.6 (-10.8, 48)	0.21	0.21
12(13)-EpOME	$PM_{2.5}$	lag2	-23.2 (-52.5, 6.1)	0.13	5.3 (-18.4, 29.1)	0.66	0.66
12(13)-EpOME	$PM_{2.5}$	lag3	-14.3 (-47.6, 19.1)	0.41	13.7 (-7.8, 35.2)	0.21	0.21
12(13)-EpOME	$PM_{2.5}$	lag4	-31.8 (-64.2, 0.6)	0.06	10.7 (-11.7, 33.2)	0.35	0.35
12(13)-EpOME	$PM_{2.5}$	lag04	-38.5 (-77.1, 0.1)	0.06	23.8 (-13.3, 61)	0.21	0.21
13-HOTrE	$PM_{2.5}$	lag0	-16.1 (-49.1, 17)	0.35	-12.2 (-51, 26.6)	0.54	0.54
13-HOTrE	$PM_{2.5}$	lag1	-11 (-38.6, 16.5)	0.44	0 (-35.2, 35.2)	1.00	1.00
13-HOTrE	$PM_{2.5}$	lag2	4.8 (-28.4, 37.9)	0.78	21.1 (-6, 48.2)	0.13	0.13
13-HOTrE	$PM_{2.5}$	lag3	2.8 (-32.2, 37.9)	0.88	20.2 (-2.9, 43.3)	0.09	**0.09**
13-HOTrE	$PM_{2.5}$	lag4	-4 (-40, 32.1)	0.83	20.9 (-5.4, 47.2)	0.12	0.12
13-HOTrE	$PM_{2.5}$	lag04	-9.8 (-53.7, 34.2)	0.67	30.4 (-14.3, 75)	0.18	0.18
14-HDHA and 11-HDoHE	$PM_{2.5}$	lag0	21.4 (-16.1, 59)	0.27	-39.4 (-85.4, 6.7)	0.09	**0.09**
14-HDHA and 11-HDoHE	$PM_{2.5}$	lag1	32.6 (-2.7, 67.8)	0.08	6.9 (-34.5, 48.4)	0.74	0.74
14-HDHA and 11-HDoHE	$PM_{2.5}$	lag2	2.4 (-38.5, 43.4)	0.91	-30.8 (-63.8, 2.3)	0.07	**0.07**
14-HDHA and 11-HDoHE	$PM_{2.5}$	lag3	1.9 (-44.8, 48.7)	0.94	-10.5 (-40.5, 19.5)	0.49	0.49
14-HDHA and 11-HDoHE	$PM_{2.5}$	lag4	1.9 (-44.5, 48.2)	0.94	-18.2 (-50.1, 13.8)	0.26	0.26
14-HDHA and 11-HDoHE	$PM_{2.5}$	lag04	31.6 (-23.9, 87.2)	0.27	-40.9 (-94.3, 12.5)	0.13	0.13
17-HDHA	$PM_{2.5}$	lag0	**42.6 (5.1, 80.2)**	**0.03**	-25.9 (-70, 18.1)	0.25	0.25
17-HDHA	$PM_{2.5}$	lag1	**66.9 (31.3, 102.5)**	**0.001**	28.3 (-10.8, 67.3)	0.16	0.16
17-HDHA	$PM_{2.5}$	lag2	18.3 (-21.3, 58)	0.37	-2.9 (-33.7, 27.9)	0.85	0.85
17-HDHA	$PM_{2.5}$	lag3	21.4 (-22.9, 65.6)	0.35	-8.9 (-35.4, 17.7)	0.51	0.51
17-HDHA	$PM_{2.5}$	lag4	15.4 (-26.8, 57.5)	0.48	-5.7 (-35, 23.5)	0.70	0.70
17-HDHA	$PM_{2.5}$	lag04	**56.8 (3.9, 109.6)**	**0.04**	-8.2 (-55.8, 39.3)	0.73	0.73
12-HEPE	$PM_{2.5}$	lag0	-2.3 (-26.9, 22.2)	0.85	-1.3 (-30.3, 27.7)	0.93	0.93
12-HEPE	$PM_{2.5}$	lag1	-4.3 (-25.6, 17)	0.69	-4.3 (-27.9, 19.3)	0.72	0.72
12-HEPE	$PM_{2.5}$	lag2	0.7 (-21.3, 22.6)	0.95	**20.8 (3.1, 38.6)**	**0.02**	**0.02**
12-HEPE	$PM_{2.5}$	lag3	0.2 (-25.9, 26.3)	0.99	10.1 (-6.7, 26.9)	0.24	0.24
12-HEPE	$PM_{2.5}$	lag4	-6.3 (-32, 19.4)	0.63	10.9 (-6.8, 28.5)	0.23	0.23
12-HEPE	$PM_{2.5}$	lag04	-3.7 (-35.3, 27.9)	0.82	21 (-8.7, 50.7)	0.17	0.17
18-HEPE	$PM_{2.5}$	lag0	-6.3 (-25.1, 12.4)	0.51	11.1 (-11.6, 33.8)	0.34	0.34
18-HEPE	$PM_{2.5}$	lag1	-4.4 (-20.8, 12)	0.60	7.1 (-12.7, 26.9)	0.48	0.48
18-HEPE	$PM_{2.5}$	lag2	-5.6 (-24.3, 13.1)	0.56	**16.5 (1.3, 31.6)**	**0.03**	**0.03**
18-HEPE	$PM_{2.5}$	lag3	-6.1 (-27.9, 15.7)	0.59	0.7 (-13.5, 14.8)	0.93	0.93
18-HEPE	$PM_{2.5}$	lag4	-4.3 (-26.1, 17.5)	0.70	-9.3 (-24.4, 5.9)	0.23	0.23
18-HEPE	$PM_{2.5}$	lag04	-11 (-37.4, 15.3)	0.42	7.4 (-18.3, 33.2)	0.57	0.57

Note: Shaded sections using gray, light blue, and blue-gray represent oxylipins derived from omega-6 polyunsaturated fatty acids including arachidonic acid (AA), dihomo-γ-linoleic acid (DGLA), and linoleic

acid (LA), respectively. Similarly, yellow, orange, and green represent oxylipins derived from omega-3 polyunsaturated fatty acids (n-3 FA) including alpha-linolenic acid (ALA), docosahexaenoic acid (DHA), and eicosapentaenoic acid (EPA), respectively. Linear mixed-effects models with random participants effects were used to examine the associations between air pollution with oxylipins in the high and low n-3 groups and test the between-group differences. Models were adjusted for age, sex, race, marital status, education, and body mass index (BMI), long-term and seasonal trends, day of the week, temperature, and relative humidity. Values [with 95% confidence interval (95%CI)] are percentage changes for an interquartile range increase in 24-hr average $PM_{2.5}$ concentrations in the low and high n-3 groups. Statistical significance was set at a two-sided $p < 0.05$ for the effects of air pollution and at a two-sided $p < 0.1$ for the interaction with n-3 FA groups.

Supplemental Table S2. Associations between O_3 exposure and changes in blood levels of oxylipins.

Oxylipins (pg/mL)	Exposure	Lag	Low n-3 group	P value	High n-3 group	P value	P value between groups
12-HETE	O_3	lag0	35.8 (-32.6, 104.1)	0.31	5.9 (-35.7, 47.4)	0.78	0.44
12-HETE	O_3	lag1	7.3 (-56.2, 70.8)	0.82	4.2 (-41, 49.3)	0.86	0.93
12-HETE	O_3	lag2	-31.1 (-89.1, 26.9)	0.30	34.5 (-10.3, 79.2)	0.13	0.06
12-HETE	O_3	lag3	-6.5 (-61.8, 48.9)	0.82	2.3 (-48, 52.5)	0.93	0.82
12-HETE	O_3	lag4	-3.8 (-45.4, 37.7)	0.86	**-51.8 (-89.8, -13.8)**	**0.01**	0.12
12-HETE	O_3	lag04	-28.7 (-143.1, 85.7)	0.63	-12.7 (-89, 63.6)	0.74	0.77
15-HETE	O_3	lag0	23.7 (-26.9, 74.4)	0.36	-11 (-42, 19.9)	0.49	0.23
15-HETE	O_3	lag1	-21.8 (-69.4, 25.8)	0.38	-6.1 (-41.6, 29.3)	0.73	0.56
15-HETE	O_3	lag2	-21 (-66.9, 24.9)	0.38	22 (-13.6, 57.5)	0.23	0.09
15-HETE	O_3	lag3	-0.1 (-37.2, 37)	1.00	-13.3 (-48.6, 22.1)	0.46	0.61
15-HETE	O_3	lag4	-0.3 (-32.1, 31.6)	0.99	**-35.4 (-63.1, -7.6)**	**0.01**	0.10
15-HETE	O_3	lag04	-24.4 (-107.4, 58.7)	0.57	-39.8 (-96.4, 16.8)	0.17	0.69
12-HHTrE	O_3	lag0	-32.9 (-95.5, 29.7)	0.31	16.6 (-29.4, 62.6)	0.48	0.17
12-HHTrE	O_3	lag1	42.4 (-18.8, 103.6)	0.18	-3.2 (-50.4, 44)	0.90	0.18
12-HHTrE	O_3	lag2	-47.7 (-108.1, 12.8)	0.13	5.2 (-41, 51.4)	0.83	**0.09**
12-HHTrE	O_3	lag3	-21.1 (-71.6, 29.4)	0.42	-9.3 (-57.8, 39.3)	0.71	0.71
12-HHTrE	O_3	lag4	-14.8 (-58.7, 29.2)	0.51	6.2 (-33.8, 46.3)	0.76	0.40
12-HHTrE	O_3	lag04	-47.8 (-152.3, 56.7)	0.38	-6.1 (-83.8, 71.7)	0.88	0.31
5,6-DiHETrE	O_3	lag0	30.7 (-15.4, 76.8)	0.20	12.1 (-18.5, 42.7)	0.44	0.48
5,6-DiHETrE	O_3	lag1	-16.7 (-60, 26.5)	0.45	-4.8 (-37.5, 27.8)	0.77	0.63
5,6-DiHETrE	O_3	lag2	-7 (-49.2, 35.2)	0.75	-11.3 (-44.4, 21.9)	0.51	0.86
5,6-DiHETrE	O_3	lag3	-14.5 (-49.7, 20.7)	0.43	-15.3 (-51.5, 20.9)	0.41	0.97
5,6-DiHETrE	O_3	lag4	-7.8 (-38.2, 22.7)	0.62	-17 (-44.6, 10.7)	0.23	0.63
5,6-DiHETrE	O_3	lag04	-28.2 (-103.8, 47.4)	0.47	-26.5 (-80.9, 27.8)	0.34	0.96
11,12-DiHETrE	O_3	lag0	-23.7 (-64.2, 16.8)	0.26	-19.8 (-49.6, 10)	0.19	0.87
11,12-DiHETrE	O_3	lag1	-6.5 (-47.6, 34.6)	0.76	4.6 (-27.8, 37)	0.78	0.61
11,12-DiHETrE	O_3	lag2	-27.6 (-67.5, 12.3)	0.18	18.6 (-11.8, 49.1)	0.23	0.03
11,12-DiHETrE	O_3	lag3	-7 (-41, 26.9)	0.69	9.2 (-23.4, 41.9)	0.58	0.45
11,12-DiHETrE	O_3	lag4	-17.5 (-46.9, 12)	0.25	-11.5 (-38.3, 15.3)	0.40	0.72
11,12-DiHETrE	O_3	lag04	-58.2 (-127.5, 11.1)	0.11	-24.2 (-75.8, 27.3)	0.36	0.22
14,15-DiHETrE	O_3	lag0	34 (-26.1, 94.1)	0.27	-17.2 (-60.1, 25.7)	0.43	0.14
14,15-DiHETrE	O_3	lag1	37.5 (-24.4, 99.5)	0.24	20.5 (-28.4, 69.5)	0.41	0.61
14,15-DiHETrE	O_3	lag2	-1.1 (-64.6, 62.4)	0.97	31.7 (-17.6, 80.9)	0.21	0.33
14,15-DiHETrE	O_3	lag3	11.3 (-40.3, 62.9)	0.67	13.7 (-35.6, 63.1)	0.59	0.94
14,15-DiHETrE	O_3	lag4	18.7 (-25.9, 63.3)	0.42	-22.3 (-64.4, 19.9)	0.30	0.12
14,15-DiHETrE	O_3	lag04	64.8 (-42.8, 172.5)	0.24	20.5 (-60.9, 101.9)	0.62	0.30
TXB2	O_3	lag0	-3 (-65.3, 59.2)	0.92	-17.6 (-62.1, 26.9)	0.44	0.68
TXB2	O_3	lag1	-7.8 (-66.7, 51)	0.80	-10.6 (-57.1, 36)	0.66	0.93
TXB2	O_3	lag2	22.2 (-38.2, 82.5)	0.48	2.8 (-43.9, 49.6)	0.91	0.54
TXB2	O_3	lag3	20.2 (-28.5, 69)	0.42	30.2 (-16.4, 76.9)	0.20	0.75
TXB2	O_3	lag4	-14.2 (-56.9, 28.6)	0.52	1.2 (-39.2, 41.7)	0.95	0.53
TXB2	O_3	lag04	12.5 (-90, 114.9)	0.81	6 (-71.4, 83.5)	0.88	0.87
15-HETrE	O_3	lag0	-2.5 (-62.7, 57.6)	0.93	-21.2 (-64.1, 21.8)	0.33	0.58
15-HETrE	O_3	lag1	24.5 (-32, 81.1)	0.40	11 (-33.6, 55.6)	0.63	0.65
15-HETrE	O_3	lag2	3 (-55.4, 61.4)	0.92	-3.3 (-48.6, 41.9)	0.89	0.84
15-HETrE	O_3	lag3	-3.5 (-48.2, 41.2)	0.88	-45.7 (-88.5, -3)	0.04	0.15
15-HETrE	O_3	lag4	10.4 (-30.8, 51.6)	0.62	2.7 (-38.6, 44.1)	0.90	0.76
15-HETrE	O_3	lag04	1.6 (-96.4, 99.6)	0.97	-19.2 (-93.5, 55.2)	0.61	0.60
9-HODE	O_3	lag0	71 (-20.4, 162.5)	0.14	37.3 (-19.6, 94.2)	0.20	0.52
9-HODE	O_3	lag1	-19.5 (-106, 67.1)	0.66	36.6 (-26.3, 99.6)	0.25	0.26
9-HODE	O_3	lag2	**-96.7 (-173, -20.4)**	**0.02**	-12.4 (-72.6, 47.7)	0.69	0.06
9-HODE	O_3	lag3	46.1 (-24, 116.2)	0.20	-32.5 (-102.4, 37.3)	0.36	0.11
9-HODE	O_3	lag4	44.1 (-16.3, 104.5)	0.16	-35.4 (-89.6, 18.7)	0.20	**0.04**
9-HODE	O_3	lag04	48.6 (-106.1, 203.4)	0.54	11.8 (-95.1, 118.7)	0.83	0.61
13-HODE	O_3	lag0	-65.1 (-278.6, 148.4)	0.55	-83.5 (-260.9, 94)	0.36	0.88
13-HODE	O_3	lag1	4.8 (-201.9, 211.4)	0.96	1.8 (-162, 165.6)	0.98	0.98
13-HODE	O_3	lag2	-62.6 (-273.8, 148.6)	0.56	-23.8 (-185.6, 137.9)	0.77	0.72
13-HODE	O_3	lag3	-71.7 (-240.7, 97.2)	0.41	108.1 (-55.4, 271.6)	0.20	**0.09**
13-HODE	O_3	lag4	22.9 (-128.1, 174)	0.77	34.3 (-103.5, 172.1)	0.63	0.90
13-HODE	O_3	lag04	-92.5 (-458.7, 273.8)	0.62	-17.3 (-290.2, 255.6)	0.90	0.59
9-OxoODE	O_3	lag0	10.3 (-23.2, 43.8)	0.55	-11.7 (-32, 8.6)	0.26	0.25
9-OxoODE	O_3	lag1	-8.4 (-36.5, 19.7)	0.56	**-28.1 (-48.2, -7.9)**	**0.01**	0.22
9-OxoODE	O_3	lag2	9.2 (-18.8, 37.1)	0.52	-8.9 (-30.9, 13.2)	0.43	0.27
9-OxoODE	O_3	lag3	15.8 (-9.3, 40.9)	0.22	-12 (-35.8, 11.8)	0.32	0.12
9-OxoODE	O_3	lag4	6 (-15.5, 27.5)	0.59	-6.8 (-25.4, 11.8)	0.48	0.37

Supplemental Table 2 continued

9-OxoODE	O_3	lag04	18.8 (-32.8, 70.4)	0.48	-29.8 (-63.7, 4)	0.08	**0.05**
13-OxoODE	O_3	lag0	-34 (-99.7, 31.8)	0.32	**-62 (-111.1, -13)**	**0.01**	0.45
13-OxoODE	O_3	lag1	-26.4 (-91.5, 38.7)	0.43	**-54.5 (-106, -3)**	**0.04**	0.42
13-OxoODE	O_3	lag2	-30.5 (-100, 39)	0.39	-32.9 (-86.2, 20.5)	0.23	0.95
13-OxoODE	O_3	lag3	7.7 (-47.3, 62.8)	0.78	-42.9 (-96, 10.2)	0.11	0.15
13-OxoODE	O_3	lag4	11 (-38.4, 60.4)	0.67	-12.4 (-57.7, 33)	0.59	0.41
13-OxoODE	O_3	lag04	-84.9 (-194.4, 24.5)	0.14	**-110.7 (-192.4, -29.1)**	**0.01**	0.55
9,10-DiHOME	O_3	lag0	116.8 (-21.4, 255.1)	0.11	45.3 (-55.2, 145.8)	0.38	0.36
9,10-DiHOME	O_3	lag1	-20.6 (-157.3, 116.1)	0.77	28.6 (-76.7, 133.9)	0.59	0.51
9,10-DiHOME	O_3	lag2	**-134.4 (-265.7, -3)**	**0.05**	-43.1 (-145.5, 59.4)	0.41	0.21
9,10-DiHOME	O_3	lag3	31 (-79.5, 141.5)	0.59	-34.3 (-140.9, 72.3)	0.53	0.35
9,10-DiHOME	O_3	lag4	21.1 (-70.9, 113.1)	0.66	-79.6 (-164.1, 4.9)	0.06	0.06
9,10-DiHOME	O_3	lag04	20.4 (-210, 250.8)	0.86	-44.8 (-216.6, 127)	0.61	0.47
9(10)-EpOME	O_3	lag0	9.6 (-46.4, 65.6)	0.74	-3.1 (-39.4, 33.2)	0.87	0.69
9(10)-EpOME	O_3	lag1	-6.2 (-53.1, 40.7)	0.80	**-51.3 (-86.2, -16.3)**	**0.00**	**0.09**
9(10)-EpOME	O_3	lag2	-30.6 (-74.1, 13)	0.18	**-59.7 (-93.8, -25.6)**	**0.00**	0.24
9(10)-EpOME	O_3	lag3	7.8 (-32.2, 47.8)	0.71	-36.1 (-75.7, 3.4)	0.07	0.11
9(10)-EpOME	O_3	lag4	14.7 (-21.5, 50.9)	0.43	-6.3 (-39.1, 26.5)	0.71	0.35
9(10)-EpOME	O_3	lag04	-27.6 (-111, 55.8)	0.52	**-69.8 (-129, -10.5)**	**0.02**	0.26
12(13)-EpOME	O_3	lag0	-6.3 (-65.7, 53.1)	0.84	28.6 (-12.1, 69.3)	0.17	0.31
12(13)-EpOME	O_3	lag1	16.2 (-41.1, 73.6)	0.58	-7.5 (-52.7, 37.7)	0.74	0.43
12(13)-EpOME	O_3	lag2	0.4 (-58.9, 59.7)	0.99	-10.3 (-55.7, 35.1)	0.66	0.73
12(13)-EpOME	O_3	lag3	-30 (-76, 16)	0.21	-17.5 (-62.6, 27.5)	0.45	0.68
12(13)-EpOME	O_3	lag4	-0.1 (-42.5, 42.3)	1.00	0.6 (-38, 39.2)	0.98	0.98
12(13)-EpOME	O_3	lag04	-26.6 (-128.8, 75.6)	0.61	-13.6 (-88, 60.8)	0.72	0.77
13-HOTrE	O_3	lag0	-31.5 (-100.4, 37.5)	0.38	-3 (-51.5, 45.5)	0.90	0.47
13-HOTrE	O_3	lag1	7.4 (-55.8, 70.6)	0.82	18.7 (-31.6, 69.1)	0.47	0.74
13-HOTrE	O_3	lag2	-13.3 (-77.2, 50.5)	0.68	21.6 (-27.7, 70.9)	0.39	0.30
13-HOTrE	O_3	lag3	3.2 (-49.4, 55.8)	0.91	-1.5 (-52.2, 49.2)	0.95	0.89
13-HOTrE	O_3	lag4	-17 (-61.4, 27.4)	0.46	-33.6 (-75.6, 8.4)	0.12	0.53
13-HOTrE	O_3	lag04	-38 (-147.5, 71.6)	0.50	-14.3 (-95.8, 67.1)	0.73	0.61
14-HDHA and 11-HDoHE	O_3	lag0	-31.9 (-117.7, 53.9)	0.47	43.7 (-14.4, 101.8)	0.14	0.13
14-HDHA and 11-HDoHE	O_3	lag1	-10.7 (-92.4, 70.9)	0.80	41.4 (-20.5, 103.2)	0.19	0.25
14-HDHA and 11-HDoHE	O_3	lag2	-38 (-113.8, 37.8)	0.33	**64.9 (6.3, 123.6)**	**0.03**	**0.02**
14-HDHA and 11-HDoHE	O_3	lag3	-27.6 (-95.3, 40.1)	0.43	11.5 (-55.9, 78.9)	0.74	0.40
14-HDHA and 11-HDoHE	O_3	lag4	-30 (-89.1, 29)	0.33	-6.8 (-60.3, 46.7)	0.80	0.52
14-HDHA and 11-HDoHE	O_3	lag04	-67.6 (-204.7, 69.6)	0.34	45.7 (-53.8, 145.1)	0.37	**0.06**
17-HDHA	O_3	lag0	85.6 (-16.6, 187.7)	0.11	30.1 (-27, 87.2)	0.30	0.34
17-HDHA	O_3	lag1	20.5 (-55.7, 96.7)	0.60	65.5 (12.9, 118)	0.01	0.30
17-HDHA	O_3	lag2	-22.6 (-96.6, 51.3)	0.55	29.5 (-27.5, 86.5)	0.31	0.23
17-HDHA	O_3	lag3	14.8 (-50.1, 79.8)	0.66	35.8 (-21.9, 93.5)	0.22	0.64
17-HDHA	O_3	lag4	17 (-41.7, 75.6)	0.57	14.3 (-36.7, 65.2)	0.58	0.95
17-HDHA	O_3	lag04	104.5 (-37.3, 246.4)	0.16	**117.4 (25.8, 209)**	**0.01**	0.85
12-HEPE	O_3	lag0	0.5 (-46.2, 47.2)	0.98	-3.4 (-34.6, 27.7)	0.83	0.88
12-HEPE	O_3	lag1	3.3 (-43.2, 49.8)	0.89	-20.9 (-55.2, 13.4)	0.23	0.36
12-HEPE	O_3	lag2	7.5 (-35.2, 50.2)	0.73	**-37.1 (-70.6, -3.6)**	**0.03**	**0.07**
12-HEPE	O_3	lag3	5.6 (-32.8, 44.1)	0.78	-14.4 (-52.7, 24)	0.46	0.46
12-HEPE	O_3	lag4	16.4 (-16.2, 49)	0.33	22.5 (-6.6, 51.6)	0.13	0.77
12-HEPE	O_3	lag04	23.5 (-58.6, 105.7)	0.58	-12.4 (-69.3, 44.6)	0.67	0.35
18-HEPE	O_3	lag0	1.3 (-38, 40.6)	0.95	-2.4 (-30.9, 26.2)	0.87	0.87
18-HEPE	O_3	lag1	11 (-26.2, 48.3)	0.56	-3.7 (-33, 25.6)	0.81	0.46
18-HEPE	O_3	lag2	-3.2 (-41.5, 35.1)	0.87	-9.5 (-38.7, 19.8)	0.53	0.75
18-HEPE	O_3	lag3	-6.2 (-37.2, 24.8)	0.70	0.8 (-29, 30.6)	0.96	0.72
18-HEPE	O_3	lag4	2.8 (-24.2, 29.9)	0.84	9.8 (-14.8, 34.5)	0.43	0.65
18-HEPE	O_3	lag04	2.2 (-63, 67.4)	0.95	-1.3 (-49.6, 47.1)	0.96	0.89

Note: Shaded sections using gray, light blue, and blue-gray represent oxylipins derived from omega-6 polyunsaturated fatty acids including arachidonic acid (AA), dihomo-γ-linoleic acid (DGLA), and linoleic acid (LA), respectively. Similarly, yellow, orange, and green represent oxylipins derived from omega-3 polyunsaturated fatty acids (n-3 FA) including alpha-linolenic acid (ALA), docosahexaenoic acid (DHA), and eicosapentaenoic acid (EPA), respectively. Linear mixed-effects models with random participants

effects were used to examine the associations between air pollution with oxylipins in the high and low n-3 groups and test the between-group differences. Models were adjusted for age, sex, race, marital status, education, and body mass index (BMI), long-term and seasonal trends, day of the week, temperature, and relative humidity. Values [with 95% confidence interval (95%CI)] are percentage changes for an interquartile range increase in maximum 8-hr average O_3 concentrations in the low and high n-3 groups. Statistical significance was set at a two-sided $p < 0.05$ for the effects of air pollution and at a two-sided $p < 0.1$ for the interaction with n-3 FA groups.

Supplemental Table S3. Associations between NO_2 exposure and changes in blood levels of oxylipins.

Oxylipins (pg/mL)	Exposure	Lag	Low n-3 group	P value	High n-3 group	P value	P value between groups
12-HETE	NO_2	lag0	23.1 (-7.7, 54)	0.15	**-25 (-41.9, -8)**	**0.004**	**0.01**
12-HETE	NO_2	lag1	0.9 (-41.9, 43.7)	0.97	-30.7 (-64.3, 2.9)	0.07	0.24
12-HETE	NO_2	lag2	-0.5 (-32.6, 31.6)	0.98	-1.5 (-51.8, 48.7)	0.95	0.97
12-HETE	NO_2	lag3	-30.2 (-77.4, 17.1)	0.22	-8 (-35.3, 19.3)	0.57	0.47
12-HETE	NO_2	lag4	13.5 (-16, 43)	0.37	**-30.7 (-52.2, -9.2)**	**0.01**	**0.01**
12-HETE	NO_2	lag04	11.9 (-50.6, 74.4)	0.71	**-75 (-116.4, -33.6)**	**0.00**	**0.02**
15-HETE	NO_2	lag0	-17.8 (-37.1, 1.5)	0.08	-10.8 (-23.8, 2.2)	0.10	0.55
15-HETE	NO_2	lag1	-15.4 (-36.3, 5.5)	0.16	-8.2 (-35.2, 18.8)	0.55	0.63
15-HETE	NO_2	lag2	-1.7 (-25, 21.5)	0.88	7.8 (-27.4, 43)	0.66	0.62
15-HETE	NO_2	lag3	-3.5 (-33.8, 26.7)	0.82	0.3 (-20.6, 21.3)	0.97	0.84
15-HETE	NO_2	lag4	3.6 (-19.9, 27.1)	0.77	-8.2 (-26.1, 9.8)	0.37	0.43
15-HETE	NO_2	lag04	-33.3 (-73.5, 6.9)	0.11	-27.9 (-64, 8.1)	0.13	0.82
12-HHTrE	NO_2	lag0	-11.1 (-37.2, 15.1)	0.41	-14.6 (-33.3, 4.1)	0.13	0.82
12-HHTrE	NO_2	lag1	-3.4 (-30.1, 23.3)	0.80	-24.7 (-59.6, 10.2)	0.17	0.27
12-HHTrE	NO_2	lag2	-4.6 (-35.2, 26)	0.77	-21.4 (-67.8, 24.9)	0.36	0.47
12-HHTrE	NO_2	lag3	9.5 (-30.8, 49.7)	0.65	-0.9 (-29.2, 27.3)	0.95	0.68
12-HHTrE	NO_2	lag4	9.9 (-20.5, 40.3)	0.53	1 (-21.8, 23.7)	0.93	0.63
12-HHTrE	NO_2	lag04	-14.5 (-67.2, 38.2)	0.59	-38.1 (-89.7, 13.5)	0.15	0.41
5,6-DiHETrE	NO_2	lag0	-7.3 (-26.2, 11.6)	0.46	-5.5 (-18.5, 7.6)	0.41	0.87
5,6-DiHETrE	NO_2	lag1	-4.5 (-24, 15.1)	0.65	-7.5 (-33.1, 18)	0.56	0.83
5,6-DiHETrE	NO_2	lag2	-8.4 (-30.3, 13.5)	0.45	-2.3 (-34.9, 30.2)	0.89	0.73
5,6-DiHETrE	NO_2	lag3	-21.6 (-49.2, 5.9)	0.13	4 (-15, 23)	0.68	0.14
5,6-DiHETrE	NO_2	lag4	-5.6 (-27.4, 16.1)	0.61	5.4 (-11.1, 21.9)	0.52	0.42
5,6-DiHETrE	NO_2	lag04	-25.5 (-64.4, 13.3)	0.21	-9.2 (-44.4, 25.9)	0.61	0.45
11,12-DiHETrE	NO_2	lag0	-14.4 (-31.9, 3.1)	0.12	**-13.7 (-26.1, -1.3)**	**0.03**	0.95
11,12-DiHETrE	NO_2	lag1	0.5 (-18.5, 19.4)	0.96	-6.7 (-31.5, 18.1)	0.60	0.60
11,12-DiHETrE	NO_2	lag2	-7.2 (-28.5, 14.1)	0.51	-11.3 (-43.7, 21)	0.49	0.80
11,12-DiHETrE	NO_2	lag3	11.5 (-16.2, 39.3)	0.42	5.3 (-14.2, 24.7)	0.60	0.72
11,12-DiHETrE	NO_2	lag4	-1.8 (-23.5, 20)	0.87	-14.1 (-30.4, 2.2)	0.09	0.36
11,12-DiHETrE	NO_2	lag04	-21.6 (-57.3, 14.1)	0.24	**-37.3 (-72.1, -2.5)**	**0.04**	0.43
14,15-DiHETrE	NO_2	lag0	-16.5 (-45.4, 12.4)	0.27	-12.4 (-32.6, 7.8)	0.23	0.81
14,15-DiHETrE	NO_2	lag1	-15.6 (-46.7, 15.5)	0.33	-2.5 (-40.4, 35.4)	0.90	0.53
14,15-DiHETrE	NO_2	lag2	27.6 (-4.1, 59.4)	0.10	20.5 (-27.8, 68.8)	0.41	0.77
14,15-DiHETrE	NO_2	lag3	26.8 (-17.4, 70.9)	0.24	-2.6 (-32.2, 27)	0.86	0.28
14,15-DiHETrE	NO_2	lag4	22.9 (-9.7, 55.5)	0.18	-21.3 (-47.2, 4.5)	0.11	0.04
14,15-DiHETrE	NO_2	lag04	3.5 (-54.9, 61.9)	0.91	-29.7 (-85.7, 26.2)	0.30	0.32
TXB2	NO_2	lag0	7.1 (-18.2, 32.4)	0.59	**-23.1 (-40.6, -5.6)**	**0.01**	**0.05**
TXB2	NO_2	lag1	-2 (-30.5, 26.6)	0.89	-24.4 (-59.1, 10.3)	0.17	0.25
TXB2	NO_2	lag2	-6.8 (-37, 23.4)	0.66	-10 (-55.9, 35.9)	0.67	0.89
TXB2	NO_2	lag3	5.2 (-36, 46.4)	0.81	8.3 (-19.3, 35.9)	0.56	0.90
TXB2	NO_2	lag4	18.5 (-13.1, 50.1)	0.26	13.2 (-11.9, 38.3)	0.30	0.79
TXB2	NO_2	lag04	9.1 (-44.6, 62.8)	0.74	-24.4 (-75.9, 27)	0.35	0.27
15-HETrE	NO_2	lag0	2.6 (-25.9, 31)	0.86	-2.6 (-21.4, 16.2)	0.79	0.76
15-HETrE	NO_2	lag1	25.7 (-2.3, 53.7)	0.08	14.3 (-18.7, 47.3)	0.40	0.54
15-HETrE	NO_2	lag2	0.7 (-29.8, 31.1)	0.97	-9.1 (-56.1, 37.9)	0.70	0.67
15-HETrE	NO_2	lag3	14.6 (-29.3, 58.5)	0.52	6.2 (-21.3, 33.8)	0.66	0.76
15-HETrE	NO_2	lag4	9.7 (-21.9, 41.4)	0.55	-14.5 (-38.6, 9.6)	0.24	0.23
15-HETrE	NO_2	lag04	25.9 (-26.6, 78.4)	0.34	-1.4 (-51.8, 48.9)	0.96	0.36
9-HODE	NO_2	lag0	-18.2 (-56.1, 19.7)	0.35	11.2 (-14.7, 37)	0.40	0.20
9-HODE	NO_2	lag1	-7.8 (-46.9, 31.3)	0.70	27.9 (-23.1, 78.9)	0.28	0.21
9-HODE	NO_2	lag2	-17 (-59.2, 25.2)	0.43	34.2 (-29.7, 98.2)	0.29	0.14
9-HODE	NO_2	lag3	-33.3 (-87.3, 20.6)	0.23	-20.9 (-57.8, 16)	0.27	0.71
9-HODE	NO_2	lag4	-22.4 (-64.6, 19.9)	0.31	-20.9 (-53.3, 11.5)	0.21	0.96
9-HODE	NO_2	lag04	-66.7 (-142.3, 8.9)	0.09	-15 (-83.5, 53.4)	0.67	0.24
13-HODE	NO_2	lag0	-23 (-117.9, 71.9)	0.64	-23.5 (-90.3, 43.3)	0.49	0.99
13-HODE	NO_2	lag1	-71.4 (-164.5, 21.8)	0.14	4.9 (-116.4, 126.2)	0.94	0.25
13-HODE	NO_2	lag2	-17.8 (-133.2, 97.5)	0.76	14.4 (-150, 178.9)	0.86	0.71
13-HODE	NO_2	lag3	-75.1 (-215.9, 65.6)	0.30	38.6 (-57.9, 135.2)	0.43	0.20
13-HODE	NO_2	lag4	-77.9 (-188.7, 32.9)	0.18	17 (-65.6, 99.5)	0.69	0.17
13-HODE	NO_2	lag04	-162.5 (-355.2, 30.1)	0.11	-33.2 (-211.2, 144.8)	0.71	0.21
9-OxoODE	NO_2	lag0	8 (-5.1, 21.1)	0.24	-0.6 (-9.5, 8.2)	0.89	0.28
9-OxoODE	NO_2	lag1	3 (-10.8, 16.8)	0.67	0 (-17.8, 17.9)	1.00	0.77
9-OxoODE	NO_2	lag2	12.7 (-1.4, 26.9)	0.09	-9.6 (-31, 11.9)	0.38	**0.06**
9-OxoODE	NO_2	lag3	14.1 (-4.1, 32.4)	0.14	-10.2 (-22.7, 2.2)	0.11	**0.04**
9-OxoODE	NO_2	lag4	13.8 (-0.4, 27.9)	0.06	5.8 (-5.1, 16.7)	0.30	0.38

Supplemental Table 3 continued

9-OxoODE	NO_2	lag04	**32.6 (7.3, 57.9)**	**0.02**	1.3 (-21.6, 24.2)	0.91	**0.04**
13-OxoODE	NO_2	lag0	-17.7 (-48.6, 13.2)	0.27	3 (-19.1, 25.2)	0.79	0.25
13-OxoODE	NO_2	lag1	-8.7 (-39.6, 22.3)	0.59	-14.7 (-55.2, 25.8)	0.48	0.79
13-OxoODE	NO_2	lag2	-11.5 (-47.3, 24.2)	0.53	-29.2 (-81.9, 23.5)	0.28	0.51
13-OxoODE	NO_2	lag3	17.8 (-27.9, 63.5)	0.45	-5.3 (-38.9, 28.3)	0.76	0.42
13-OxoODE	NO_2	lag4	2.2 (-34.1, 38.4)	0.91	-19.3 (-46.8, 8.1)	0.17	0.34
13-OxoODE	NO_2	lag04	-25.9 (-87.8, 35.9)	0.42	-33.2 (-93.1, 26.6)	0.28	0.83
9,10-DiHOME	NO_2	lag0	25.4 (-31.9, 82.7)	0.39	**54.9 (14.6, 95.3)**	**0.01**	0.38
9,10-DiHOME	NO_2	lag1	-7.7 (-69, 53.6)	0.81	26.1 (-54.1, 106.3)	0.52	0.44
9,10-DiHOME	NO_2	lag2	-9 (-80.3, 62.2)	0.80	32.1 (-72.8, 137.1)	0.55	0.44
9,10-DiHOME	NO_2	lag3	-70.1 (-158, 17.8)	0.13	-8.6 (-73, 55.9)	0.79	0.27
9,10-DiHOME	NO_2	lag4	-31.5 (-103.7, 40.7)	0.40	-0.9 (-56.1, 54.4)	0.97	0.49
9,10-DiHOME	NO_2	lag04	-19.5 (-140.8, 101.8)	0.75	68.5 (-48.2, 185.2)	0.25	0.18
9(10)-EpOME	NO_2	lag0	10.5 (-11.7, 32.7)	0.36	4.5 (-10.8, 19.8)	0.57	0.66
9(10)-EpOME	NO_2	lag1	-1.2 (-24.3, 21.9)	0.92	5 (-25.2, 35.2)	0.75	0.71
9(10)-EpOME	NO_2	lag2	-18.9 (-43.6, 5.8)	0.14	-7.7 (-45.2, 29.8)	0.69	0.57
9(10)-EpOME	NO_2	lag3	-14.8 (-46.2, 16.5)	0.36	-20.3 (-42.1, 1.5)	0.07	0.78
9(10)-EpOME	NO_2	lag4	6.9 (-18.6, 32.4)	0.60	9.1 (-10.3, 28.6)	0.36	0.89
9(10)-EpOME	NO_2	lag04	-7.3 (-52.8, 38.2)	0.75	0.7 (-41.6, 43)	0.97	0.76
12(13)-EpOME	NO_2	lag0	-25.1 (-50.2, 0)	0.06	2.2 (-15.7, 20)	0.81	**0.07**
12(13)-EpOME	NO_2	lag1	-5.6 (-29.8, 18.6)	0.65	**43.6 (11.8, 75.4)**	**0.01**	**0.01**
12(13)-EpOME	NO_2	lag2	-20.9 (-50.1, 8.3)	0.17	-6.1 (-50.4, 38.2)	0.79	0.51
12(13)-EpOME	NO_2	lag3	-0.5 (-38.8, 37.7)	0.98	17.3 (-9.5, 44.2)	0.21	0.46
12(13)-EpOME	NO_2	lag4	-27.3 (-57, 2.3)	0.08	-15.4 (-37.6, 6.8)	0.18	0.51
12(13)-EpOME	NO_2	lag04	**-55.2 (-105.1, -5.3)**	**0.04**	3.6 (-43.4, 50.6)	0.88	**0.04**
13-HOTrE	NO_2	lag0	-10.2 (-37.3, 16.9)	0.47	17.4 (-2.6, 37.4)	0.09	**0.09**
13-HOTrE	NO_2	lag1	-5.6 (-33.8, 22.6)	0.70	19.5 (-17.3, 56.3)	0.30	0.23
13-HOTrE	NO_2	lag2	-16.4 (-48.7, 15.8)	0.32	11.4 (-38.3, 61.1)	0.65	0.27
13-HOTrE	NO_2	lag3	9.8 (-31.8, 51.3)	0.65	6.8 (-26, 39.6)	0.68	0.91
13-HOTrE	NO_2	lag4	5.6 (-26, 37.1)	0.73	21.5 (-3.3, 46.3)	0.09	0.42
13-HOTrE	NO_2	lag04	-1.2 (-55.7, 53.2)	0.96	49.3 (-3.3, 102)	0.07	0.10
14-HDHA and 11-HDoHE	NO_2	lag0	14.4 (-21.7, 50.5)	0.44	-9.5 (-34.8, 15.9)	0.46	0.27
14-HDHA and 11-HDoHE	NO_2	lag1	16.8 (-20.1, 53.7)	0.38	5 (-43.5, 53.4)	0.84	0.66
14-HDHA and 11-HDoHE	NO_2	lag2	-1.9 (-42.2, 38.4)	0.93	-41.7 (-102.9, 19.5)	0.18	0.21
14-HDHA and 11-HDoHE	NO_2	lag3	7.2 (-43.9, 58.3)	0.78	33.2 (-2.3, 68.7)	0.07	0.42
14-HDHA and 11-HDoHE	NO_2	lag4	3.5 (-37, 44)	0.87	-33.4 (-64.1, -2.7)	0.03	0.15
14-HDHA and 11-HDoHE	NO_2	lag04	27.7 (-44.4, 99.7)	0.46	-23.9 (-92.8, 44.9)	0.50	0.20
17-HDHA	NO_2	lag0	**50.7 (19.4, 82)**	**0.00**	6.6 (-13.3, 26.5)	0.52	0.02
17-HDHA	NO_2	lag1	**70.7 (39.6, 101.8)**	**0.00**	**64.6 (24.2, 105.1)**	**0.00**	0.79
17-HDHA	NO_2	lag2	20.6 (-20.7, 62)	0.33	21.5 (-39.8, 82.9)	0.49	0.98
17-HDHA	NO_2	lag3	-8.9 (-57.8, 40)	0.72	**38.1 (4.7, 71.6)**	**0.03**	0.13
17-HDHA	NO_2	lag4	-8.8 (-47.7, 30.1)	0.66	0 (-31.1, 31.1)	1.00	0.72
17-HDHA	NO_2	lag04	**100.9 (36.3, 165.5)**	**0.00**	**66.3 (10.5, 122.1)**	**0.02**	0.37
12-HEPE	NO_2	lag0	-7.6 (-27.8, 12.6)	0.47	-9.9 (-23.6, 3.7)	0.15	0.84
12-HEPE	NO_2	lag1	-13.8 (-34, 6.5)	0.19	-20.3 (-46.8, 6.1)	0.13	0.65
12-HEPE	NO_2	lag2	-6.5 (-30.2, 17.1)	0.59	5.8 (-28.6, 40.2)	0.74	0.52
12-HEPE	NO_2	lag3	6.5 (-22.5, 35.5)	0.66	15 (-5.9, 35.9)	0.16	0.65
12-HEPE	NO_2	lag4	-0.3 (-23.5, 23)	0.98	6.9 (-11.3, 25.1)	0.46	0.63
12-HEPE	NO_2	lag04	-17 (-58.9, 24.9)	0.43	-7.8 (-45.4, 29.7)	0.68	0.70
18-HEPE	NO_2	lag0	-3.3 (-17.9, 11.3)	0.66	-8.7 (-18.9, 1.6)	0.10	0.54
18-HEPE	NO_2	lag1	-2.1 (-17.4, 13.2)	0.79	-1.7 (-21.8, 18.4)	0.87	0.97
18-HEPE	NO_2	lag2	2.1 (-14.5, 18.8)	0.80	17.4 (-7.9, 42.7)	0.18	0.24
18-HEPE	NO_2	lag3	2.4 (-19.8, 24.6)	0.83	4.3 (-11.2, 19.9)	0.59	0.89
18-HEPE	NO_2	lag4	-0.1 (-17.4, 17.3)	0.99	-9.8 (-22.9, 3.2)	0.14	0.37
18-HEPE	NO_2	lag04	-8.8 (-38.6, 21.1)	0.57	-15.4 (-43.9, 13)	0.29	0.69

Note: Shaded sections using gray, light blue, and blue-gray represent oxylipins derived from omega-6 polyunsaturated fatty acids including arachidonic acid (AA), dihomo-γ-linoleic acid (DGLA), and linoleic acid (LA), respectively. Similarly, yellow, orange, and green represent oxylipins derived from omega-3 polyunsaturated fatty acids (n-3 FA) including alpha-linolenic acid (ALA), docosahexaenoic acid (DHA), and eicosapentaenoic acid (EPA), respectively. Linear mixed-effects models with random participants

effects were used to examine the associations between air pollution with oxylipins in the high and low n-3 groups and test the between-group differences. Models were adjusted for age, sex, race, marital status, education, and body mass index (BMI), long-term and seasonal trends, day of the week, temperature, and relative humidity. Values [with 95% confidence interval (95%CI)] are percentage changes for an interquartile range increase in 24-hr average NO_2 concentrations in the low and high n-3 groups. Statistical significance was set at a two-sided $p < 0.05$ for the effects of air pollution and at a two-sided $p < 0.1$ for the interaction with n-3 FA groups.
